# Alkaline Ethanol Oxidation Reaction on Carbon Supported Ternary PdNiBi Nanocatalyst using Modified Instant Reduction Synthesis Method

**DOI:** 10.1007/s12678-019-00577-8

**Published:** 2020-01-03

**Authors:** Bernd Cermenek, Boštjan Genorio, Thomas Winter, Sigrid Wolf, Justin G. Connell, Michaela Roschger, Ilse Letofsky-Papst, Norbert Kienzl, Brigitte Bitschnau, Viktor Hacker

**Affiliations:** 1 Institute of Chemical Engineering and Environmental Technology, Fuel Cell Systems Group, Graz University of Technology, NAWI Graz, Inffeldgasse 25/C, 8010 Graz, Austria; 2 Faculty of Chemistry and Chemical Technology, University of Ljubljana, Večna pot 113, 1000 Ljubljana, Slovenia; 3 Materials Science Division, Argonne National Laboratory, 9700 South Cass Avenue, Lemont, IL 60439 USA; 4 Institute for Electron Microscopy and Nanoanalysis and Center for Electron Microscopy, Graz University of Technology, NAWI Graz, Steyrergasse 17, 8010 Graz, Austria; 5 Bioenergy 2020+ GmbH, Inffeldgasse 21/B, 8010 Graz, Austria; 6 Institute of Physical and Theoretical Chemistry, Graz University of Technology, Stremayrgasse 9, 8010 Graz, Austria

**Keywords:** Pd_85_Ni_10_Bi_5_ nanocatalyst, Modified instant reduction synthesis method, Ethanol oxidation reaction activity, Structure, Morphology, Alkaline direct ethanol fuel cell

## Abstract

Direct ethanol fuel cells (DEFC) still lack active and efficient electrocatalysts for the alkaline ethanol oxidation reaction (EOR). In this work, a new instant reduction synthesis method was developed to prepare carbon supported ternary PdNiBi nanocatalysts with improved EOR activity. Synthesized catalysts were characterized with a variety of structural and compositional analysis techniques in order to correlate their morphology and surface chemistry with electrochemical performance. The modified instant reduction synthesis results in well-dispersed, spherical Pd_85_Ni_10_Bi_5_ nanoparticles on Vulcan XC72R support (Pd_85_Ni_10_Bi_5_/C^(II-III)^), with sizes ranging from 3.7 ± 0.8 to 4.7 ± 0.7 nm. On the other hand, the common instant reduction synthesis method leads to significantly agglomerated nanoparticles (Pd_85_Ni_10_Bi_5_/C^(I)^). EOR activity and stability of these three different carbon supported PdNiBi anode catalysts with a nominal atomic ratio of 85:10:5 were probed via cyclic voltammetry and chronoamperometry using the rotating disk electrode method. Pd_85_Ni_10_Bi_5_/C^(II)^ showed the highest electrocatalytic activity (150 mA⋅cm^−2^; 2678 mA⋅mg^−1^) with low onset potential (0.207 V) for EOR in alkaline medium, as compared to a commercial Pd/C and to the other synthesized ternary nanocatalysts Pd_85_Ni_10_Bi_5_/C^(I)^ and Pd_85_Ni_10_Bi_5_/C^(III)^. This new synthesis approach provides a new avenue to developing efficient, carbon supported ternary nanocatalysts for future energy conversion devices.

**Electronic supplementary material:**

The online version of this article (10.1007/s12678-019-00577-8) contains supplementary material, which is available to authorized users.

**Graphical Abstract:**
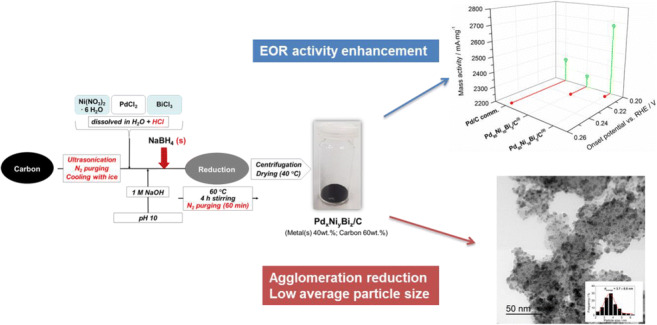
The modified instant reduction method for synthesis of ternary Pd_85_Ni_10_Bi_5_/C^(II)^ nanocatalyst using Vulcan XC72R as carbon support initiates an agglomeration reduction, provides low average particle size, and enables enhanced activity for the alkaline ethanol oxidation reaction (EOR) compared to the common instant reduction method and to a commercial Pd/C catalyst.

## Introduction

Demand for renewable energy has been recently increasing due to economic and environmental requirements, with fuel cells identified as a key technology for the clean energy industry of the future [1, 2]. Critical technical barriers that restrict the commercialization of fuel cells include performance (activity), durability (stability), and costs of state-of-the-art catalysts [3, 4]. Direct ethanol fuel cells (DEFC) have attracted attention in the last decades due to their robustness, low cost, and environmental compatibility [5, 6]. In addition, ethanol is a carbon dioxide neutral fuel with a relatively high energy density (8.01 kWh⋅kg^−1^) comparable to gasoline (11 kWh⋅kg^−1^) that can be directly converted into electricity in a fuel cell [4, 7]. Commercialization of DEFCs is currently still hampered by slow kinetics of the anode electrochemical reactions and by the ethanol crossover through the membrane. The latter is responsible for occurrence of a mixed potential which results in a reduction of the cathode potential and subsequently a decrease in power density of the DEFC system. The use of an anion exchange membrane (AEM) in alkaline DEFCs results in a reversed ion conduction mechanism relative to acidic DEFCs, which reduces the challenges of fuel crossover and additionally simplifies the water management [8].

Nevertheless, slow kinetics for the EOR remains the major challenge for DEFC development. Complete ethanol oxidation is a 12e^−^ reaction that leads to a formation of CO_2_ and water. State-of-the-art catalyst systems lead to an incomplete, 4e^−^ oxidation reaction resulting in CH_3_COOH (acetate, CH_3_COO^−^) formation. Pt is the most used catalyst for alcohol oxidation in acidic media, but high cost, low availability, and prevalent poisoning by adsorbed CO-like intermediates formed during ethanol oxidation limit its application. The use of alkaline electrolytes significantly improves the kinetics of the ethanol oxidation reaction (EOR) and of the oxygen reduction reaction (ORR) [8, 9] and allows the replacement of expensive Pt catalysts by more abundant, inexpensive, and non-noble metal-containing catalysts [4, 6, 10]. Currently, Pd/C is the most suitable catalyst for the DEFC in alkaline solution, with significantly higher EOR activity relative to Pt/C [11]. This can be attributed to the more oxophilic character of, and improved C-C bond cleavage on, the Pd surface relative to Pt. Further, Pd is known to have higher stability and is less susceptible to poisoning effects than Pt [5, 11, 12].

Chatenet et al. [39] investigated the stability/durability/degradation of two commercial Pd/C catalysts firstly after a short and a long accelerated stress test (AST) of 150 and 1000 CV cycles in acidic and alkaline media and secondly in alkaline electrolytes in the absence/presence of strong reducing agents (hydrogen, hydrazine borane). It was found that the degradation of these two Pd/C catalysts in the acidic electrolyte is high, but their stability in the alkaline electrolyte is higher than Pt/C catalysts. In addition, the carbon supported Pd nanoparticles in alkaline media with strong reducing agents have an insignificantly lower stability than without, whereby the instability of large particles is lower than that of smaller ones. Another study in Chatenet and colleagues [40] also showed that after an AST of 150 CV cycles with different characterization methods, the Pd/C catalyst is less active against CO oxidation to carbonates and CO_2_, leading to a lower degree of detachment of Pd nanoparticles from the carbon carrier Vulcan XC72 compared to PtRu/C and Pt/C catalysts. This means that the corrosion resistance of the carbon carrier Vulcan XC72 in the presence of Pd nanoparticles is greater than that of PtRu and Pt nanoparticles, indicating that the Pd/C catalyst is more stable in alkaline media than the other two.

The reaction mechanism of EOR on Pd-based catalysts is still not fully understood. Wang et al. as well as other research groups [12–14] describe the generally accepted mechanism of EOR on Pd in alkaline media by Eqs. 1–5:

1 $$ {\mathrm{Pd}}^0+{\mathrm{OH}}^{-}\leftrightarrows {\mathrm{Pd}}^0--{\mathrm{OH}}_{\mathrm{ads}}+{\mathrm{e}}^{-} $$

2 $$ {\mathrm{Pd}}^0+{\mathrm{CH}}_3{\mathrm{CH}}_2\mathrm{OH}\leftrightarrows \mathrm{Pd}\hbox{--} {\left({\mathrm{CH}}_3{\mathrm{CH}}_2\mathrm{OH}\right)}_{\mathrm{ads}} $$

3 $$ \mathrm{Pd}\hbox{--} {\left({\mathrm{CH}}_3{\mathrm{CH}}_2\mathrm{O}\mathrm{H}\right)}_{\mathrm{ads}}+3\ {\mathrm{OH}}^{-}\leftrightarrows \mathrm{Pd}\hbox{--} {\left({\mathrm{COCH}}_3\right)}_{\mathrm{ads}}+3\ {\mathrm{H}}_2\mathrm{O}+{3\mathrm{e}}^{-} $$

4 $$ \mathrm{Pd}\hbox{--} {\left({\mathrm{COCH}}_3\right)}_{\mathrm{ads}}+\mathrm{Pd}\hbox{--} {\mathrm{OH}}_{\mathrm{ads}}\leftrightarrows \mathrm{Pd}\hbox{--} {\left({\mathrm{CH}}_3\mathrm{COOH}\right)}_{\mathrm{ads}}+{\mathrm{Pd}}^0 $$

5 $$ \mathrm{Pd}\hbox{--} {\left({\mathrm{CH}}_3\mathrm{COOH}\right)}_{\mathrm{ads}}+{\mathrm{OH}}^{-}\leftrightarrows {\mathrm{Pd}}^0+{\mathrm{CH}}_3{\mathrm{COO}}^{-}+{\mathrm{H}}_2\mathrm{O} $$

The above mechanism points out the importance of the OH^−^ ions, and the conversion of the acyl intermediates to acetate. Liang et al. [14] described that the exchange of the adsorbed acyl (COCH_3_)_ads_ for the adsorbed hydroxyl is the rate determining step (Eq. 4), while the dissociative adsorption of ethanol is a fast reaction [12, 15, 16].

Based on this understanding, much research has been done in recent years to improve the EOR performance of anode materials and to reduce costs by developing new synthesis methods for catalyst nanostructure preparation, specifically to alloy Pd with other metals like Ru, Pb, Sb, As, Ni, or Bi, as well as to improve the active surface area of the catalyst material on its support [5, 17–19]. The benefits of bi- and trimetallic catalysts are that the additives are usually inexpensive and act as co-catalysts that improve catalytic activity and stability [1, 8]. Ni is a particularly promising co-catalyst candidate for Pd, as Ni/Pd alloys have shifted lattice constants that usually promote synergetic effects. For example, the surface binding energy for reaction intermediates is lower, and therefore, the effect of poisoning by different adsorbents like CO or CH_x_ species is reduced [20–22]. Moreover, Ni has a high OH^−^ affinity which is crucial for the EOR as described above [2]. Further, Neto et al. [23] reported that adding Bi to Pd has a positive impact on the performance of the anode catalyst, which has been proposed to yield additional oxide and hydroxide species that increase OH^−^ adsorption on the catalyst surface, thus improving the activity for EOR [23, 24]. Cai et al. have also demonstrated that Pd has great affinity Bi^(III)^-ion adsorption, which is important in the catalyst synthesis [18]. Also important to the electrocatalytic activity of Pd catalysts is the uniform dispersion of the nanoparticles on a suitable support material [1, 8], which results in a higher active surface area and enables lower total loading subsequently lower costs [25]. It is well-known that the support material also influences the active component, particle size and distribution, morphology, and stability. As a result, the most commonly used supports are carbon-based materials (e.g., Vulcan XC72R from CABOT Corporation) that show an excellent combination of surface properties, electronic conductivity, and corrosion resistance [26, 27].

Herein, we introduce a new synthesis approach in order to enhance the EOR activity/stability and to improve the dispersion of ternary PdNiBi/C nanocatalyst on Vulcan XC72R carbon support. This method improves upon the approach developed in our previous study [28], which yielded Pd_x_Ni_y_Bi_z_/C anode catalysts with high activity and high by-product tolerance toward alkaline EOR but resulted in nanoparticles with inhomogeneous morphology that tended to agglomerate and provided a nonuniform distribution on the carbon support. The new synthesis method utilized the following modifications of the common instant reduction method: (i) using HCl instead of NaCl for dissolution of the PdCl_2_, (ii) performing the synthesis process under N_2_ inert atmosphere and ice-bath cooling, and (iii) addition of solid NaBH_4_ as a reducing agent. Catalysts prepared by our new synthesis method were fully characterized and compared to catalysts obtained by previously published method [28]. Electrochemical analysis revealed that the above modifications in the synthesis approach are crucial for improved activity and selectivity of ternary PdNiBi/C nanocatalyst toward EOR reaction. This modified approach points to a new route to developing novel catalysts for energy conversion devices of the future.

## Experimental

### Chemicals and Materials

Palladium chloride (PdCl_2_, anhydrous, 59–60% Pd basis, Aldrich), sodium chloride (NaCl, ≥ 99.5%, Carl Roth), nickel(II) nitrate hexahydrate (Ni(NO_3_)_2_⋅6H_2_O, Aldrich), bismuth(III) chloride (BiCl_3_, reagent grade, ≥ 98%, Aldrich), hydrochloric acid (HCl (aq), Carl Roth), Vulcan XC72R carbon black (CABOT Corporation, USA), sodium borohydride (NaBH_4_, purity of 97%, Alfa Aesar), 2-propanol (≥ 99.8%, Honeywell), sodium hydroxide (NaOH, Fluka), and ultrapure water (~ 18 MΩ∙cm, Barnstead NANOpure Water Purification system) were used for the development of Pd_85_Ni_10_Bi_5_ nanoparticles on the Vulcan XC72R carbon support material as anode catalysts. A commercial Pd/C (40 wt.%, Fuel Cell Store) catalyst was used as reference material.

### Anode Catalyst Synthesis

The carbon supported ternary Pd_85_Ni_10_Bi_5_ anode catalysts were synthesized by the common [28] as well as the modified instant reduction method using Vulcan XC72R as catalyst support material (catalyst composition: 60 wt.% of carbon support and 40 wt.% of metal). The modified instant method was developed based on the common instant method with the following modifications.

The precursor salt solution is prepared by dissolving PdCl_2_ salt in 10 mL of ultrapure water with additional 1.5 mL of 1 M HCl instead of NaCl. This results in a better solubility and faster dissolution time (Eq. 6).

6 $$ {\mathrm{PdCl}}_2+2\ \mathrm{HCl}\to {\mathrm{H}}_2\left[{\mathrm{PdCl}}_4\right] $$

The dissolution of Ni(NO_3_)_2_∙6H_2_O salt is carried out in 10 mL of ultrapure water with additional 1.5 mL of 1 M HCl. The precursor salt solution BiCl_3_ (aq.) is prepared similarly with a few drops of HCl [28].

The overall synthesis process is performed under N_2_ atmosphere in the ice bath. Vulcan XC72R carbon is dispersed in 60 mL of ultrapure water (without the addition of 2-propanol), and the suspension is sonicated two times for 5 min with a cycle of 0.6 and an amplitude of 40%, using an ultrasonic probe (Hilscher, UP400s). Afterward, the precursor salt solutions are added to the Vulcan XC72R carbon black dispersion under constant sonication, and the pH of the mixture is adjusted to 10 with 1 M NaOH. The addition of the reducing agent NaBH_4_ (~ 5 eq.) to the catalyst dispersion takes place in chunks (pure solid form) or dropwise in liquid form (0.6 mL of 1 M NaOH and 6 mL of ultrapure water).

**Fig. 1:**
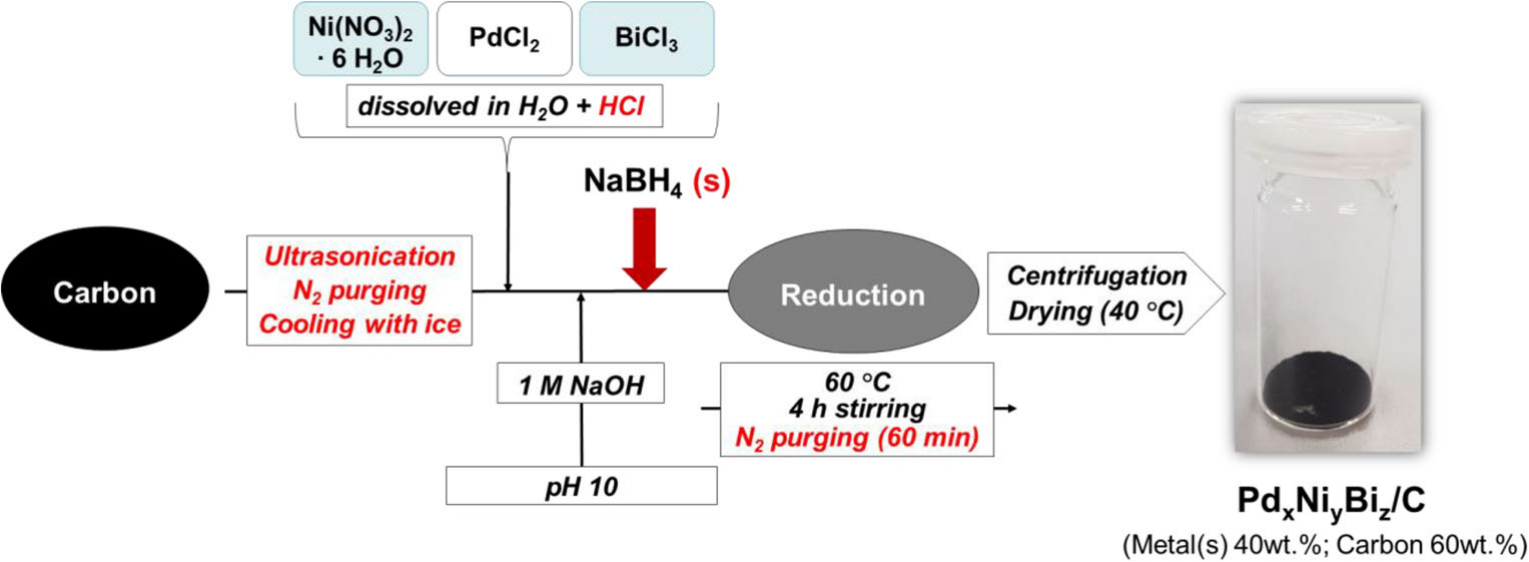
Modified instant reduction synthesis method – changes are marked red compared to the common instant reduction synthesis method described previously

The reaction mixture is vigorously stirred at 60 °C for 4 h (note: N_2_ purging is switched off after 1 h**)** to obtain the carbon supported Pd_85_Ni_10_Bi_5_/C^(II)^ (NaBH_4_(s)) and Pd_85_Ni_10_Bi_5_/C^(III)^ (NaBH_4_(l)) catalysts. After cooling to room temperature, multiple washing with ultrapure water and centrifugation is performed. Finally, the purified catalyst samples are dried at 40 °C for 24 h [28]. Figure 1 presents a schematic representation of the modified instant reduction synthesis method.

### Physicochemical Characterization

#### Inductively Coupled Plasma Optical Emission Spectrometry (ICP-OES)

The elemental compositions of two ternary Pd_85_Ni_10_Bi_5_ catalyst samples on Vulcan XC72R carbon support (Pd_85_Ni_10_Bi_5_/C^(II)^ and Pd_85_Ni_10_Bi_5_/C^(III)^) prepared by the modified instant reduction synthesis method, one ternary Pd_85_Ni_10_Bi_5_ catalyst sample on Vulcan XC72R carbon support (Pd_85_Ni_10_Bi_5_/C^(I)^) prepared by the common instant reduction synthesis method, and one commercial Pd/C catalyst sample were obtained by ICP-OES. The catalyst powders were pre-treated by microwave-assisted pressurized acid digestion using a Multiwave 3000 microwave system (Anton Paar, Graz, Austria). The catalyst samples (~ 10 mg) were digested for 25 min in 7 mL of concentrated nitric acid, 0.2 mL of HClO_4_ (Carl Roth, Karlsruhe, Germany), and 0.2 mL of hydrofluoric acid 40% (Merck, Darmstadt, Germany) at a temperature of 195 °C (heating rate: 13 °C/min) and a power of 1500 W. The digested catalysts were diluted to 25 mL with deionized water, and the ICP-OES analyses were carried out utilizing an Arcos SOP system by Spectro (Kleve, Germany). Following detection wavelengths were used for each element: Bi 223.061 nm, Ni 231.604 nm, and Pd 340.458 nm. Sample blanks and spikes were included in all preparation procedures.

#### Transmission Electron Microscopy Coupled with Energy-Dispersive X-Ray Spectroscopy (TEM-EDX)

The morphology, particle size distribution, and the chemical composition (atomic ratio) of the Pd_85_Ni_10_Bi_5_/C nanocatalysts were identically analyzed by TEM-EDX according to [28]. TEM imaging was conducted on a monochromated TF20 TEM (FEI) equipped with a Schottky field emission gun at a maximum accelerating voltage of 200 kV and an EDAX Si(Li) detector was used to record EDX spectra. The TEM grids were prepared by the standard preparation procedure for powder samples. The sample particles were suspended in alcohol and dropped onto a copper TEM grid which was covered with a holey carbon support film. The evaluation of the TEM-EDX measurements of each catalyst (*d*-spacing determinations and element quantifications) was performed by Digital Micrograph software program. For the particle size distribution and average particle diameter determination, we took 100 spherical-shaped particles of the TEM micrograph areas for Pd_85_Ni_10_Bi_5_/C^(II)^ and Pd_85_Ni_10_Bi_5_/C^(III)^ catalysts and measured the diameter of particles manually by FIJI (ImageJ)-free software program [29].

#### X-Ray Powder Diffraction (XRD)

XRD characterization of the structure for each synthesized anode catalyst was performed by means of a Bruker D8 Advance powder diffractometer with Lynxeye detector, Cu Kα X-ray source (λ = 1.54187 Å), in the range of 5° to 100° 2θ, a step size of 0.02°, and a counting time of 3 s step^−1^. The software program X’Pert HighScore plus (PANalytical) was used for determination of half-width (B_observed_), lattice parameter, and *d*-spacing using profile fit and Rietveld refinement. The average crystallite size (*L*) of located crystal structures in different catalyst powders was estimated according to Scherrer (Eq. 7) assuming spherical crystallites with cubic crystal system.

7 $$ L=\kern0.75em \frac{K\cdot \lambda }{\kern0.75em {B}_{2\mathrm{\varTheta}}\cdot \mathit{\cos}\uptheta\ } $$

*K*: Constant which is assigned to the value of 0.94 for assumption of spherical crystallites with cubic crystal system.
λ: Wavelength of the Cu Kα1 radiation (= 1.54056 Å), only Kα1 peaks were used.
B: Difference between half width (full width half maximum FWHM, peak width = B_observed_) and the device related width (= B_standard_) using the measurement of the standard material LaB_6_.

#### X-Ray Photoelectron Spectroscopy (XPS)

XPS measurements were performed using a SPECS PHOIBOS 150 Hemispherical Energy Analyzer with a monochromated Al Kα X-ray source. Indium foil was used as substrate for the sample preparation. Survey spectra were measured using a pass energy of 40 eV at a resolution of 0.2 eV step^−1^ and a total integration time of 0.1 s point^−1^. Data analysis was performed using CasaXPS software (http://www.casaxps.com/) with a Shirley-type background and 70–30 Gaussian–Lorentzian peak shapes, except for Pd metal, which was fit with an asymmetric line shape.

### Electrochemical Characterization

The prepared Pd_85_Ni_10_Bi_5_/C and commercial Pd/C nanocatalysts were ex situ characterized by means of thin film rotating disk electrode (RDE; Model AFE5T050GC from PINE Research Instrumentation) technique using a standard three electrode setup in an electrochemical glass cell (Metrohm) [28, 41]. The glassy carbon (GC)-RDE, coated with the catalyst acted as working electrode, the counter electrode, was a platinized titanium rod (Bank Elektronik – Intelligent controls GmbH), and a reversible hydrogen electrode (RHE; HydroFlex®, gaskatel) was utilized as reference electrode. A GAMRY (bi-)potentiostat (Reference 600™ Potentiostat/Galvanostat/ZRA, GAMRY Instruments Inc., Pennsylvania, USA) was used as control system.

For the preparation of the working electrode, the catalyst was transferred to a GC-RDE (Ø = 5 mm; 0.196 cm^2^) via a suspension. A mixture of 2-propanol, ultrapure water, and Nafion ionomer solution (5 wt.%, Quintech) as binder was prepared to form a homogeneous suspension of the synthesized catalysts. The slurry was sonicated two times for 5 min with a cycle of 0.6 and an amplitude of 20% using an ultrasonic probe. Before each electrochemical measurement, the RDE was polished using an Al_2_O_3_ suspension (MasterPrep® Polishing Suspension; Buehler) with a particle size of 0.05 μm and was finally rinsed with ultrapure water. Afterward, the generated catalyst ink was applied onto the GC disk by a micropipette, to achieve a loading of 56 μg_Pd_ cm^−2^ on the surface. The RDE with the catalyst ink was rotated for approx. 1.5 h at 700 rpm to uniformly dry and distribute the catalyst material. The active layer thicknesses (ALT) of the Pd/C (commercial) and Pd_85_Ni_10_Bi_5_/C^(I)-(III)^ catalysts on the GC-RDE are approx. 3 μm and 10 μm, respectively. Details for ALT calculations of the catalysts are given in the ESM.

In order to determine the electrochemical active surface area (EASA) and the EOR activity of all synthesized nanocatalysts, cyclic voltammetry (CV) measurements using RDE were recorded in nitrogen (N_2_, purity of 5.0, Air Liquide), purged 1.0 M potassium hydroxide (1.0 M KOH standard solution; FIXANAL KA 180 mL, Sigma-Aldrich), and in a mixture of 1 M KOH and 1 M EtOH (purchased from EtOH absolute, 99.999%, Aldrich). Three independent CV measurements of each catalyst to each 6 cycles were performed at 30 °C and with a scan rate of 10 mV∙s^−1^. The last cycle was used for evaluation of their electrochemical properties. The charge of the integrated reduction peak of PdO to Pd (Q_Pd_) located between 0.65 V and 0.90 V in Fig. 6a as well as 0.55–0.75 V in Fig. S10a was used for EASA determination of all catalyst samples according to Eq. 8 using further parameters, such as the assumed charge of the reduction peak PdO to Pd (${Q}_{Pd}^{\ast}\Big)$ according to literature [28], the GC area of the RDE (A_GC_ = 0.196 cm^2^), and catalyst loading of Pd on GC-RDE (c_L_ = 0.056 mg_Pd_⋅cm^−2^). The Bi oxide reduction is in the same region as the Pd oxide reduction; therefore both resulting charges were used in the calculation of EASA for the ternary Pd_85_Ni_10_Bi_5_/C catalysts as explained in [28, 34].

8 $$ EASA\ \left({cm}^2\cdot {mg}^{-1}\right)=\frac{Q_{Pd}\ \left(\mu C\right)}{\kern0.75em {Q}_{Pd}^{\ast }\ \left(\mu C\cdot {cm}^{-2}\right)\cdot {A}_{GC}\left({cm}^2\right)\cdot {c}_L\left( mg\cdot {cm}^{-2}\right)\ } $$

Also, three independent chronoamperometry (CA) measurements using RDE were carried out at a potential of 0.83 V at 30 °C for 1 h to examine the EOR stability of the catalysts. All electrochemical characterization results are given as mean value plus standard deviation. Further details about the CV measurement process are described in [28].

## Results and Discussion

### Physicochemical Characterization of Anode Catalysts

Differences in synthesis between Pd_85_Ni_10_Bi_5_/C^(I)^, Pd_85_Ni_10_Bi_5_/C^(II)^, and Pd_85_Ni_10_Bi_5_/C^(III)^ nanocatalysts are summarized in the experimental section above but are briefly discussed here again. In all cases, the common instant reduction method was modified as follows to generate Pd_85_Ni_10_Bi_5_/C^(II)^ and Pd_85_Ni_10_Bi_5_/C^(III)^ catalysts: (a) HCl was used instead of NaCl for improved dissolution of the PdCl_2_, and (b) the synthesis procedure was performed under N_2_ inert atmosphere and with ice-bath cooling. Furthermore, solid or liquid NaBH_4_ was used for the synthesis of Pd_85_Ni_10_Bi_5_/C^(II)^ and Pd_85_Ni_10_Bi_5_/C^(III)^, respectively. Pd_85_Ni_10_Bi_5_/C^(I)^ was prepared by the common instant reduction method according to [28]. The elemental composition, crystal structure, and morphology (i.e., particle size, crystallinity, and shape) of all developed carbon supported ternary PdNiBi nanoparticles on Vulcan XC72R support were comprehensively analyzed by various physicochemical characterization methods, such as ICP-OES, TEM-EDX, XRD, and XPS.

**Fig. 2:**
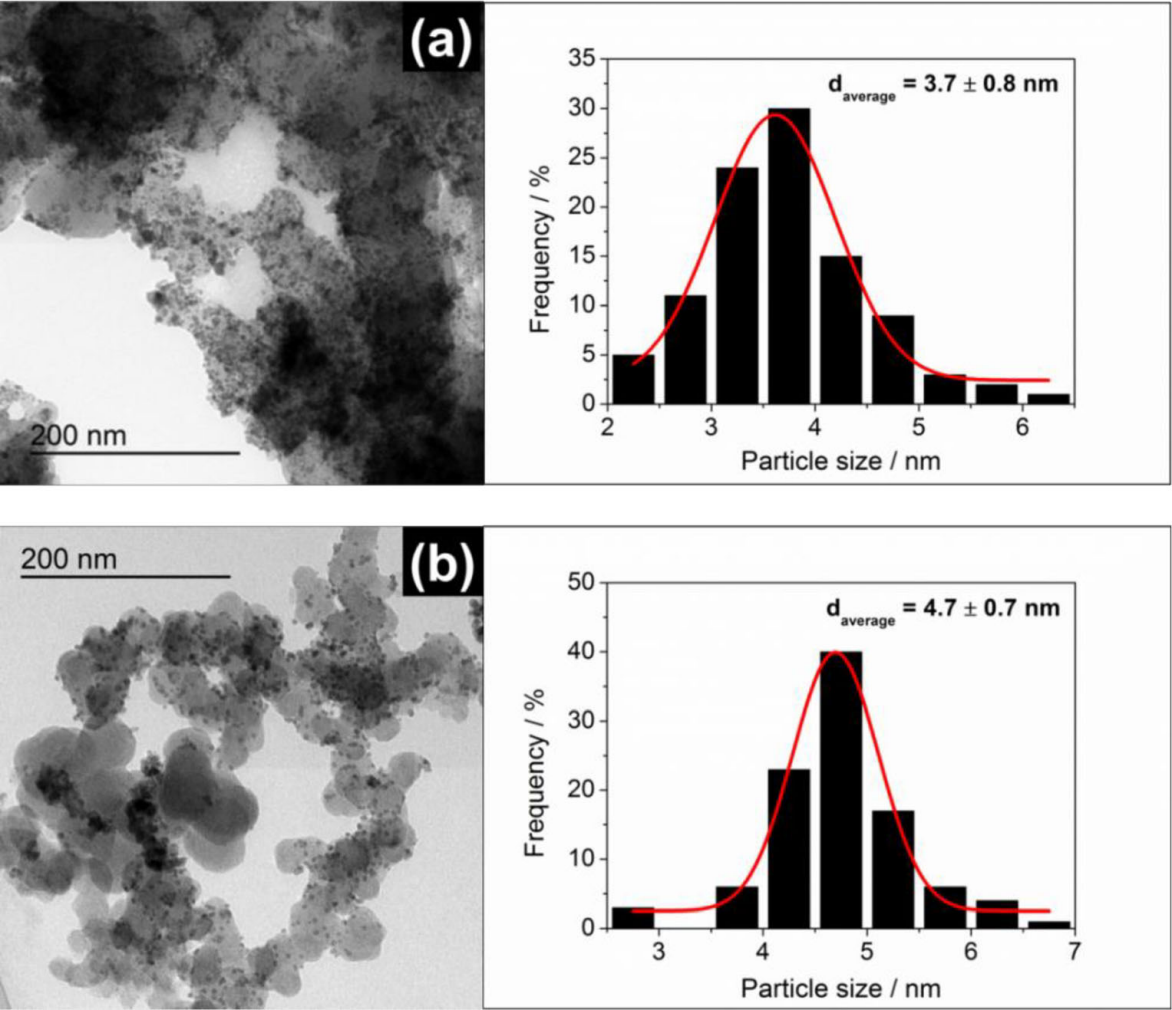
TEM micrographs and particle size distribution histograms including the determined average particle size of the (**a**) Pd_85_Ni_10_Bi_5_/C^(II)^ and (**b**) Pd_85_Ni_10_Bi_5_/C^(III)^ catalyst

**Fig. 3:**
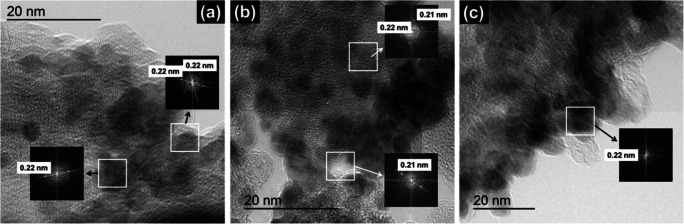
HRTEM micrographs with determined *d*-spacing of the (**a**) Pd_85_Ni_10_Bi_5_/C^(I)^, (**b**) Pd_85_Ni_10_Bi_5_/C^(II)^, and (**c**) Pd_85_Ni_10_Bi_5_/C^(III)^ catalyst

The elemental concentrations of the carbon supported Pd_x_Ni_y_Bi_z_ nanocatalysts and commercial Pd/C catalyst were determined using ICP-OES (summarized in Table S1), which revealed that atomic concentrations of synthesized anode catalysts are in agreement with targeted values for Pd_85_Ni_10_Bi_5_. As expected, quantitative analysis for commercial Pd/C catalyst revealed that material is pure with no other contaminants. The morphology, particle size distribution, and the chemical composition of each synthesized carbon supported Pd_85_Ni_10_Bi_5_ catalyst were also characterized by TEM with low and high magnification (Figs. 2 and 3, and Figs. S1, S3, and S5).

Results on Fig. 2a and b revealed that the agglomeration of the Pd_85_Ni_10_Bi_5_/C^(II)^ and Pd_85_Ni_10_Bi_5_/C^(III)^ nanoparticles on Vulcan XC72R carbon support was drastically reduced compared to the Pd_85_Ni_10_Bi_5_/C^(I)^ nanoparticles on Fig. S1 and the other previously published Pd_x_Ni_y_Bi_z_/C catalysts, which were developed by the common instant reduction synthesis method [28]. EDX spectra from chosen areas of corresponding STEM-HAADF micrograph (Figs. S2, S4, and S6) further demonstrate that the Pd, Ni, and Bi nanoparticles of all ternary Pd_85_Ni_10_Bi_5_/C catalysts are homogeneously distributed on carbon support material. The obtained TEM-EDX results of the Pd_85_Ni_10_Bi_5_/C catalysts (atomic concentrations of Pd, Ni, and Bi) are summarized in Tables S2–S4 and are in good agreement with the ICP-OES results (Table S1).

Particle size distribution and average particle diameter (*d*_average_) were evaluated from TEM images (Fig. 2a and b), with Pd_85_Ni_10_Bi_5_/C^(II)^ catalyst exhibiting smaller particles (*d*_average_ = 3.7 ± 0.8 nm) as compared to Pd_85_Ni_10_Bi_5_/C^(III)^ (*d*_average_ = 4.7 ± 0.7 nm). This difference is attributed to the use of solid NaBH_4_ during the synthesis of Pd_85_Ni_10_Bi_5_/C^(II)^ as compared to the use of liquid NaBH_4_ for Pd_85_Ni_10_Bi_5_/C^(III)^. In both cases, the nanoparticles are significantly more evenly dispersed on the carbon support, which we attribute in part to the inert N_2_ atmosphere used in synthesis. Synthesis of the Pd_85_Ni_10_Bi_5_/C^(I)^ under ambient atmosphere most likely results in the formation of various undesired oxide species, which easily tend to agglomerate with metal ions on

the carbon support material [28]. Improved dispersion of Pd_85_Ni_10_Bi_5_/C^(II)^ and Pd_85_Ni_10_Bi_5_/C^(III)^ is likely also driven by improved solubility of the precursor salt PdCl_2_ due to the substitution of HCl for the NaCl used to synthesize Pd_85_Ni_10_Bi_5_/C^(I)^. This is supported by the presence of chloride in the Pd_85_Ni_10_Bi_5_/C^(I)^ catalyst identified by TEM-EDX (Fig. S2) as well as XPS analysis (Fig. S7b), whereas no chlorides were detected in the other ternary catalyst samples (Figs. S4, S6, S7c, and S7d). The use of ice-bath cooling during the catalyst synthesis using the modified instant method likely further contributes to more homogeneous reduction of the precursor salts to metals on the carbon support material, thus resulting in better dispersibility of the PdNiBi nanoparticles.

Fast Fourier transforms of catalyst particles in the high-resolution (HR)TEM micrographs in Fig. 3 reveal intensity consistent with an interplanar spacing of ~ 0.22 nm for all ternary Pd_85_Ni_10_Bi_5_/C catalysts, which corresponds to the (111) plane spacing of the face centered cubic (*fcc*) crystalline structure of Pd.

**Fig. 4:**
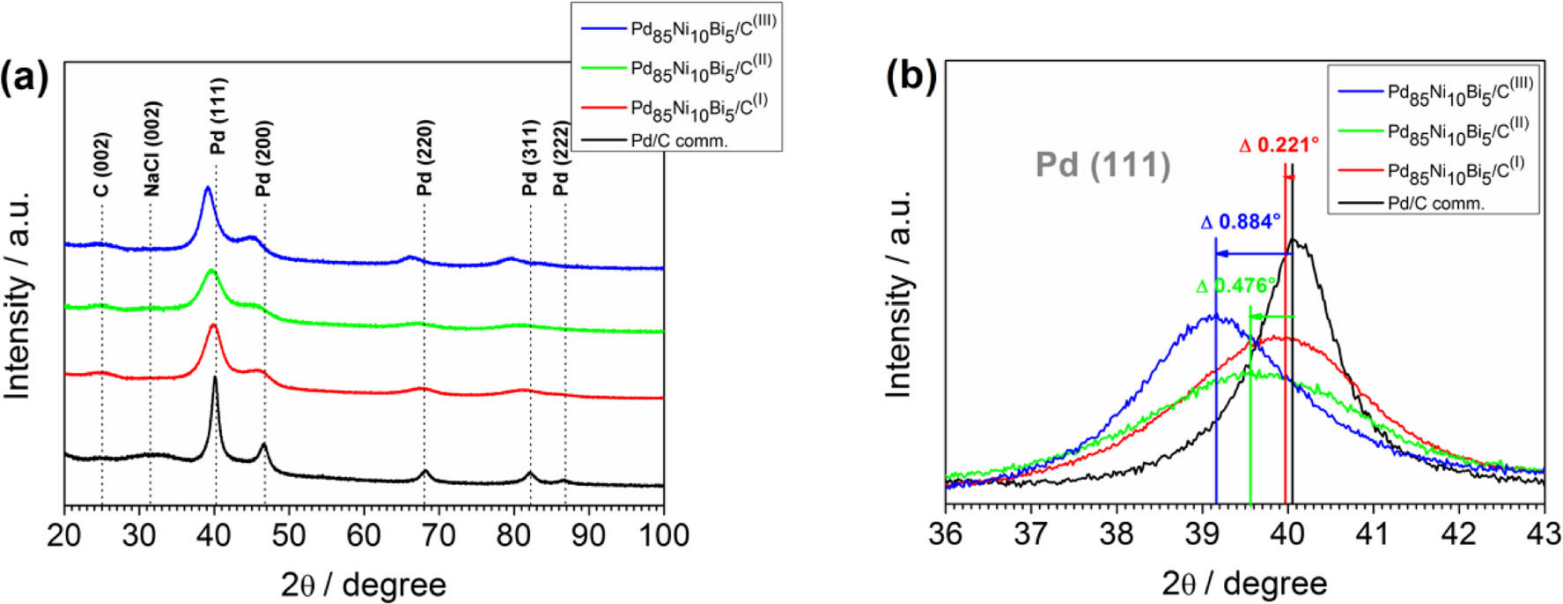
(**a**) XRD patterns of all Pd_85_Ni_10_Bi_5_/C nanocatalysts and (**b**) the corresponding position of Pd (111) diffraction peak compared to the commercial Pd/C nanocatalyst

XRD analysis of all carbon supported Pd_85_Ni_10_Bi_5_ catalysts, as well as the commercial Pd/C catalyst, also show the characteristic diffraction peaks at 2*θ* values of 40°, 47°, 68°, 82°, and 87° (Fig. 4a), which are further consistent with the (111), (200), (220), (311), and (222) spacings of the fcc Pd crystalline structure (Pd_64922-ICSD) (Tables S5–S8). The Pd (111) *d*-spacing of all ternary catalysts (Table 1) are also in good agreement with those measured from HRTEM analysis (Fig. 3). The diffraction peak at ~ 25° for all catalyst samples is identified as the (002) plane of graphite (2H_187640-ICSD) from the carbon black Vulcan XC72R (Fig. 4a and Tables S5–S8) and is consistent with our previous observation [28]. The additional diffraction peak present in the commercial Pd/C catalyst at 2θ of 31.6° (Fig. 4a and Table S5) is attributed to the (002) plane of NaCl (NaCl_655785-ICSD).

**Table 1:** Determined XRD analysis parameters of all catalyst samples. Average crystallite size estimation according to Scherrer assuming spherical crystallites with cubic crystal system of five diffraction peaks (peak pos.). Determination of half-width (FWHM; B_obs_), *d*-spacing, and lattice parameter by profile fit and Rietveld refinement, B_standard_ determined from LaB_6_

Catalysts	Lattice parameter (nm)	*d*-spacing Pd (111) (nm)	Crystallite size Pd (nm)
Pd/C comm.	a = 0.3890	0.225	9
Pd_85_Ni_10_Bi_5_/C^(I)^	a = 0.3953	0.226	3
Pd_85_Ni_10_Bi_5_/C^(II)^	a = 0.3957	0.228	4
Pd_85_Ni_10_Bi_5_/C^(III)^	a = 0.4024	0.229	5

Figure 4b shows that the typical diffraction peaks for all Pd_85_Ni_10_Bi_5_/C nanocatalysts shift to lower 2*θ* values compared to the commercial Pd/C catalyst, with peak shifts increasing in following order: Pd_85_Ni_10_Bi_5_/C^(I)^ (Δ 0.221°) < Pd_85_Ni_10_Bi_5_/C^(II)^ (Δ 0.476°) < Pd_85_Ni_10_Bi_5_/C^(III)^ (Δ 0.884°). The precise peak positions of all catalyst samples are given in the Tables S5–S12. The higher lattice parameters and *d*-spacings of all Pd_85_Ni_10_Bi_5_/C nanocatalysts compared to the commercial Pd/C catalyst (Table 1) are consistent with an expansion of Pd lattice due to the incorporation of the Ni and Bi co-catalysts to form an alloy, as has been observed before [12, 23, 28, 30–33]. The half widths of the typical diffraction peaks for all Pd_85_Ni_10_Bi_5_/C nanocatalysts are broader than for the commercial Pd/C catalyst, indicating a smaller crystallite size. Average crystallite size for all catalyst samples are estimated in Table 1 according to the Scherrer equation (Eq. 7), with crystallite size increasing as follows: Pd_85_Ni_10_Bi_5_/C^(I)^ < Pd_85_Ni_10_Bi_5_/C^(II)^ < Pd_85_Ni_10_Bi_5_/C^(III)^ < Pd/C comm. Details about the determined XRD parameters for evaluation of crystallite size (Tables S9–S12) and XRD data fitted using Rietveld refinement (Tables S5–S8) of all catalysts are provided in the Electronic Supplementary Material (ESM).

**Fig. 5:**
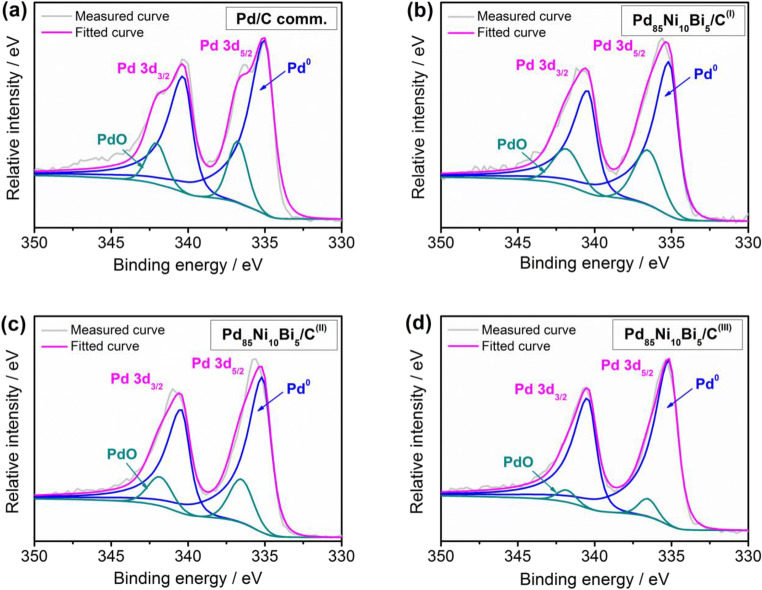
Pd 3d core level XPS spectra of the (**a**) Pd/C comm., (**b**) Pd_85_Ni_10_Bi_5_/C^(I)^, (**c**) Pd_85_Ni_10_Bi_5_/C^(II)^, and (**d**) Pd_85_Ni_10_Bi_5_/C^(III)^ catalyst

**Fig. 6:**
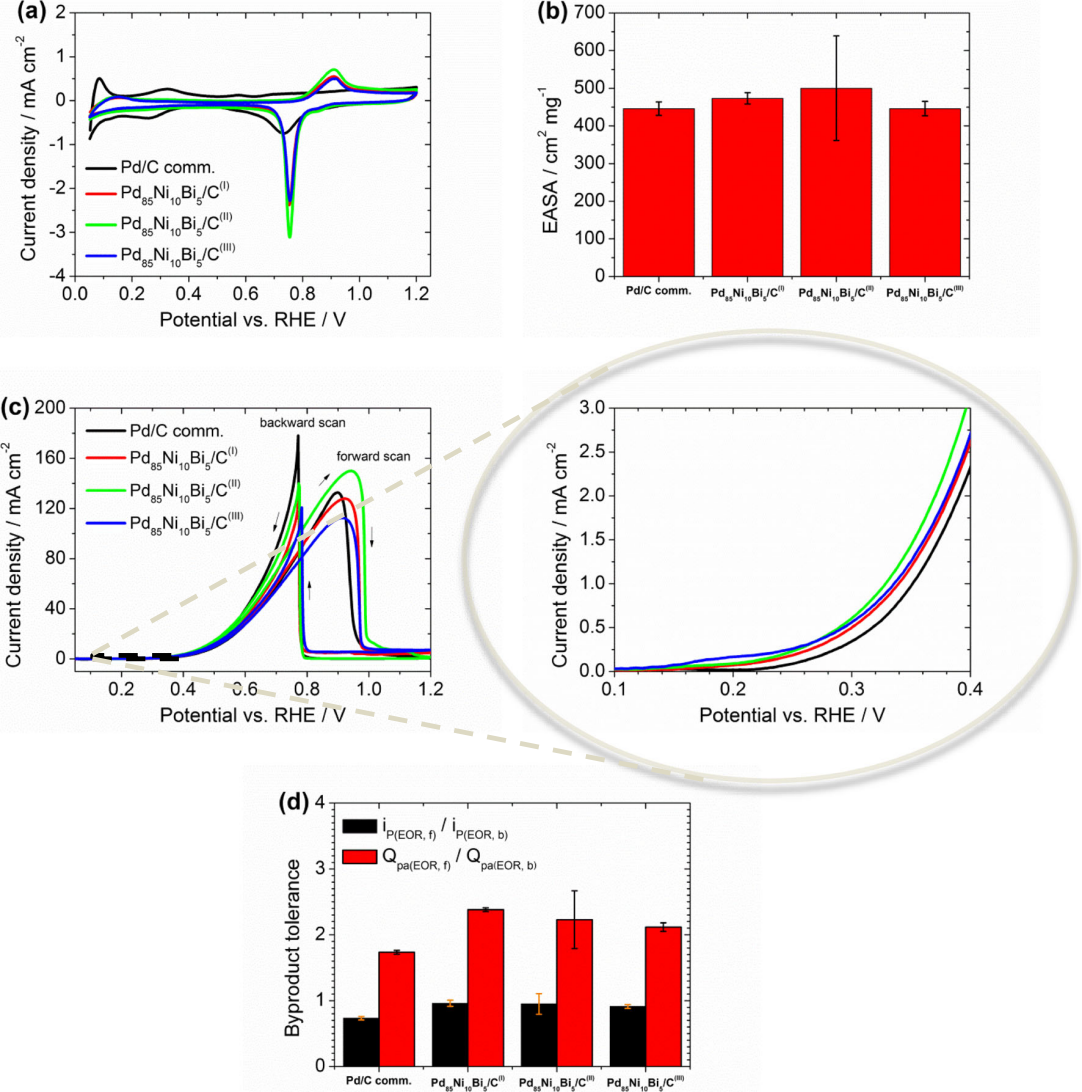
Electrochemical characterization of Pd/C comm., Pd_85_Ni_10_Bi_5_/C^(I)^, Pd_85_Ni_10_Bi_5_/C^(II)^, and Pd_85_Ni_10_Bi_5_/C^(III)^ catalysts: (a) in de-aerated 1.0 M KOH and (b) the resulting EASAs, (c) in an electrolyte mixture of 1 M KOH + 1 M EtOH, and (d) the obtained by-product tolerances resulting from EOR measurements (see (c)) – performed at 30 °C and with a scan rate of 10 mV∙s^−**1**^

The surface oxidation states, chemical compositions, and binding energies of Pd, Ni, and Bi in the synthesized Pd_85_Ni_10_Bi_5_/C catalysts and commercial Pd/C catalyst were determined by XPS. Survey spectra and extracted elemental concentrations for all catalysts are summarized in Fig. S7 and Tables S13–S17, respectively. The Pd_85_Ni_10_Bi_5_/C^(I)^ catalysts exhibit a slightly higher oxygen content as compared to the others, which is likely due to synthesis in non-inert atmosphere. Core level spectra of Pd 3d, Ni 2p, and Bi 4f were analyzed directly from the survey XPS spectra, and deconvolution of the Pd 3d, Ni 2p, and Bi 4f spectra was performed to understand the speciation of the different alloy species (see Fig. 5 and Figs. S8–S9). Two oxidation states of Pd were identified in the Pd 3d region (Fig. 5) – metallic (Pd^0^) and PdO (Pd^II^) – similar to observations in our previous study [28]. Figure 5d shows that the Pd_85_Ni_10_Bi_5_/C^(III)^ catalyst contains the most metallic Pd on the surface relative to PdO as compared with the other ternary catalysts (Fig. 5b and c), which is likely due to the excess surface Ni content on these samples (Table S17) and may also contribute to the overall lower durability of these catalysts (Fig. 6d). The Pd_85_Ni_10_Bi_5_/C^(III)^ catalyst was found to contain the highest overall Ni content of all three ternary alloys, and fitting of the Ni 2p core level (Fig. S8) reveals that Ni(OH)_2_ is the main Ni species present on all Pd_x_Ni_y_Bi_z_/C catalysts. In contrast, Pd_85_Ni_10_Bi_5_/C^(I)^ catalyst exhibits a slightly higher surface Bi content compared to the Pd_85_Ni_10_Bi_5_/C^(II)^ and Pd_85_Ni_10_Bi_5_/C^(III)^ catalysts (Table S17). Deconvolution of the Bi 4f core levels (Fig. S9) reveals similar Bi speciation for all Pd_85_Ni_10_Bi_5_/C catalysts. The primary peak is consistent with alloyed Bi metal (Bi^0^), with the peak position shifted to higher binding energy relative to pure Bi metal due to the strong binding interaction between Bi and platinum group metals [35, 36]. All alloys also contain a small, secondary peak at higher binding energy that is consistent with Bi_2_O_3_.

### Electrochemical Characterization

**Fig. 7:**
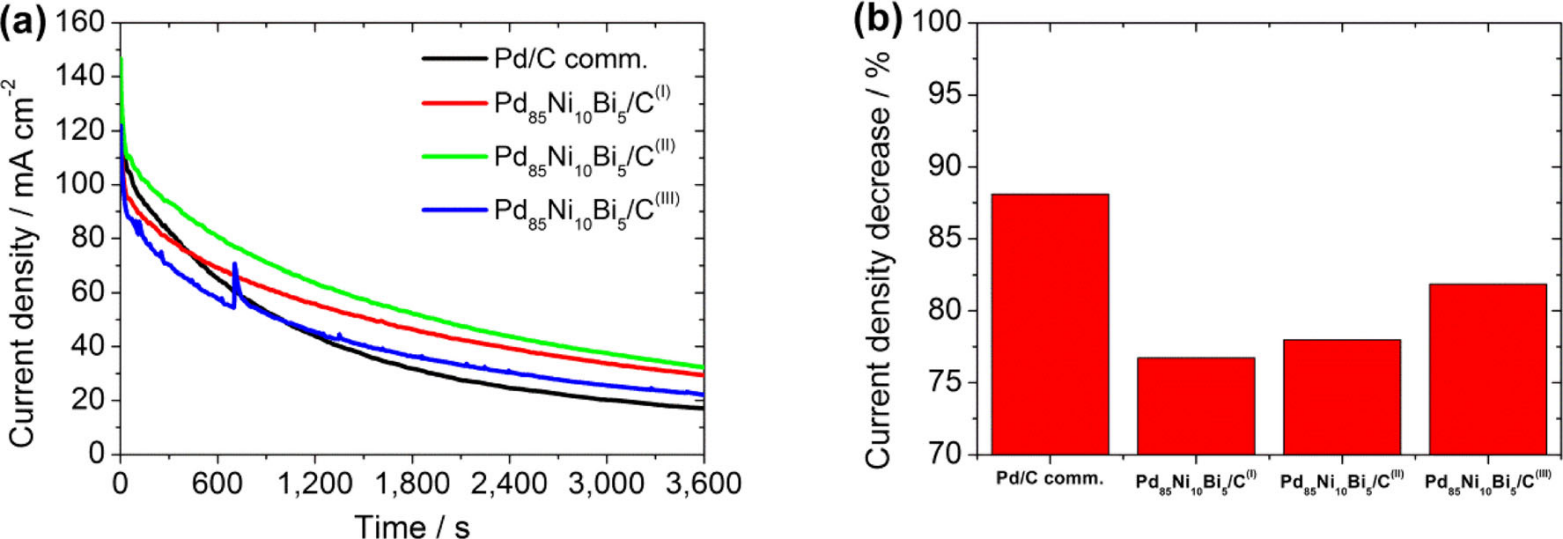
CA measurements of Pd/C comm., Pd_85_Ni_10_Bi_5_/C^(I)^, Pd_85_Ni_10_Bi_5_/C^(II)^, and Pd_85_Ni_10_Bi_5_/C^(III)^ catalysts: (a) in an electrolyte mixture of 1 M KOH + 1 M EtOH and (b) the resulting current density decrease percentage at an applied potential of 0.83 V and at 30 °C for 3600 s

The EOR performance of the various Pd_85_Ni_10_Bi_5_/C catalysts synthesized via different procedures was characterized by CV-RDE and CA-RDE and compared to the commercial Pd/C as a benchmark for the alkaline EOR in Figs. 6, 7 and S10.

Cyclic voltammograms (CVs) in the potential range of 0.05–1.2 V were recorded without rotation in de-aerated 1.0 M KOH, as well as in a mixture of 1 M KOH and 1 M EtOH at 30 °C (Fig. 6a and c), and demonstrate key differences between the different catalyst materials. As expected, in the absence of ethanol, the hydrogen adsorption and desorption peaks in the potential region between 0.05 V and 0.5 V (Fig. 6a) are suppressed for the ternary catalysts relative to commercial Pd/C. This suppression is due to the presence of bismuth, which lowers the degree of H insertion into the Pd crystal structure due to modified electronic properties as shown previously [23, 28, 34]. The characteristic peak at 0.9 V in CV of the forward scan (Fig. 6a) is attributed to the oxidation of Bi(OH)_3_ in the presence of KOH to form Bi_2_O_3_ [24, 28, 34]. Opening the potential window further to 1.5 V (Fig. S10a) reveals the oxidation of Ni(OH)_2_ to NiOOH in the positive scan and subsequent reduction of NiOOH to Ni(OH)_2_ in the negative scan for all three catalysts, indicating the presence of Ni(OH)_2_ on the surface of the catalysts that is consistent with the XPS analysis [28]. Ni redox is more pronounced on Pd_85_Ni_10_Bi_5_/C^(II)^ and Pd_85_Ni_10_Bi_5_/C^(III)^ relative to Pd_85_Ni_10_Bi_5_/C^(I)^ catalyst, which is likely due to the lower overall concentration of Ni on these samples as measured by XPS (Tables S14–S17). The Pd_85_Ni_10_Bi_5_/C^(II)^ catalyst exhibits the highest EASA compared to the other catalysts (Figs. 6b and S10b; see experimental methods for details), which may be due to a higher surface Pd content (Table S17) and/or the more homogeneously distributed metal nanoparticles with lower average particle diameter on the carbon support material (Fig. 2).

Cyclic voltammograms measured in the presence of ethanol (Fig. 6c) can be used to evaluate differences in the kinetics of the alkaline EOR on the various catalysts by measuring the slope from the onset to the peak potential. Note that here, the onset potential of the ethanol oxidation for all catalyst samples was determined at 0.1 mA⋅cm^−2^ due to electrochemical double layer charging effects, and the maximum current in the forward scan was utilized as an indicator for the EOR activity of each catalyst. In that context, Fig. 6c and the corresponding zoomed in section show that the EOR activities at low and high potential are different, which is likely due to the various catalyst surface compositions hindering or promoting the dissociation step of the ethanol oxidation. The Pd_85_Ni_10_Bi_5_/C^(II)^ catalyst shows the highest peak current density and the lowest onset potential for the alkaline EOR compared to the other catalysts, indicating these catalysts exhibit an optimal content and synergy between the Pd, Ni, and Bi species. The Pd_85_Ni_10_Bi_5_/C^(III)^ catalyst exhibits the lowest EOR activity, which is likely due to excess Ni on the catalyst surface as measured by XPS (Table S17). Indeed, the oxophilic character of Ni leads to additional adsorbed OH on the catalyst surface, which aids in the rapid oxidation of various ethanol species and enhances the by-product tolerance. However, Ni itself is inactive to alkaline EOR in the useful potential range, hindering the dissociative ethanol adsorption on the active material due to covering of active sites with excess OH, leading to overall lower activity of Pd_85_Ni_10_Bi_5_/C^(III)^ for the alkaline EOR [28, 31].

As shown in Fig. 6c, the commercial Pd/C catalyst exhibits a higher onset potential toward alkaline ethanol oxidation relative to the other catalysts, which is likely due to the faster poisoning of the Pd active sites by CO-like species at low potentials [33]. In order to assess the relative extent of surface poisoning by intermediates, the ratio of peak current density on the forward and backward scans, as well as the ratio of integrated charge, can serve as a measure of by-product tolerance (i.e., resistance to surface poisoning) [33, 37]. A comparison of these values (Fig. 6d) reveals that indeed all Pd_85_Ni_10_Bi_5_/C catalysts exhibit higher by-product tolerance relative to commercial Pd/C. The higher Bi content on the surface of Pd_85_Ni_10_Bi_5_/C^(I)^ compared to Pd_85_Ni_10_Bi_5_/C^(III)^ (Table S17) likely leads to the improved by-product tolerance on these catalysts, as Bi affords higher protection of Pd active sites against poisoning of CO-like species from the alkaline EOR [38].

The EOR stabilities of all catalyst samples were further evaluated using CA-RDE at an applied potential of 0.83 V at 30 °C for 1 h (Fig. 7) and reveal that stability decreases in the order: Pd_85_Ni_10_Bi_5_/C^(II)^ ~ Pd_85_Ni_10_Bi_5_/C^(I)^ < Pd_85_Ni_10_Bi_5_/C^(III)^ < Pd/C comm., consistent with the by-product tolerance results. The use of a teflon cell is preferred over a glass cell for the electrochemical characterization of catalysts in alkaline media due to the glass corrosion that occurs, as demonstrated by Mayrhofer et al. [42]. The catalysts are particularly deactivated in the long-term stability tests (RDE-CA measurements) by the contaminations leached out of the glass used. This could be one of the many possible reasons for the decrease of the current density after 1 h stress test at a constant potential 0.83 V. Therefore, future work will include the influence of impurities from dissolved glass on the ternary PdNiBi/C catalyst.

Table 2 summarizes the electrochemical results of all carbon supported Pd_85_Ni_10_Bi_5_/C nanocatalysts and of the commercial Pd/C catalyst.

**Table 2:** Electrochemical characterization results of carbon supported Pd_85_Ni_10_Bi_5_/C nanocatalysts developed by instant reduction synthesis method with different modifications compared to commercial Pd/C catalyst

Catalysts	EASA^a^ (cm^2^∙mg^−1^)	EASA^b^ (cm^2^∙mg^−1^)	*i*_f_^c^ (mA∙cm^−2^)	*i*_b_^c^ (mA∙cm^−2^)	*i*_f_/*i*_b_^d^	*Q*_pa,f_/*Q*_pa,b_^e^	*E*_onset_^f^ (V)	*i*_Start_^g^ (mA∙cm^−2^)	*i*_End_^g^ (mA∙cm^−2^)	*i*_D_^h^ (%)
Pd/C comm.	446 ± 18	–	132.73	178.21	0.732 ± 0.026	1.736 ± 0.029	0.249 ± 0.010	143.25	17.05	88
Pd_85_Ni_10_Bi_5_/C^(I)^	473 ± 15	746 ± 29	127.83	126.91	0.960 ± 0.047	2.381 ± 0.028	0.206 ± 0.018	125.79	29.29	77
Pd_85_Ni_10_Bi_5_/C^(II)^	500 ± 139	806 ± 239	149.96	140.04	0.951 ± 0.157	2.229 ± 0.439	0.199 ± 0.052	146.68	32.29	78
Pd_85_Ni_10_Bi_5_/C^(III)^	446 ± 19	687 ± 35	112.52	120.74	0.911 ± 0.028	2.116 ± 0.066	0.185 ± 0.027	121.92	22.11	82

^a^0.05–1.2 V; ^b^0.05–1.5 V; ^c^*i*_f_ and *i*_b_, peak current density of forward and backward scan; ^d^*i*_f_/*i*_b_, by-product tolerance using peak current density of forward and backward scan; ^e^*Q*_pa,f_/*Q*_pa,b_, by-product tolerance using the charge of the integrated peak current density area of the forward and backward scan; ^f^*E*_onset_, onset potential of the ethanol oxidation; ^g^*i*_start_ and *i*_End_, resulting current densities at an applied potential of 0.83 V after 0 s and 3600 s; ^h^*i*_D_, loss of current density after stress test at an applied potential of 0.83 V for 3600 s

## Conclusions

In this work, a new synthesis method for carbon supported PdNiBi alloy nanocatalysts has been successfully developed, with modifications in the synthesis method resulting in catalysts with smaller particle size and a more homogeneous distribution on the carbon support. The Pd_85_Ni_10_Bi_5_/C^(II)^ catalyst displays outstanding specific and mass activity (150 mA⋅cm^−2^; 2678 mA⋅mg^−1^) with a low onset potential (0.207 V) for the alkaline EOR. It was found that the synthesis of the Pd_85_Ni_10_Bi_5_/C^(II)^ catalyst in N_2_ atmosphere, the use of HCl, and the addition of solid NaBH_4_ result in the highest EOR performance among all catalyst samples, with activity decreasing as: Pd_85_Ni_10_Bi_5_/C^(II)^ > Pd/C comm. > Pd_85_Ni_10_Bi_5_/C^(I)^ > Pd_85_Ni_10_Bi_5_/C^(III)^. We further demonstrate that the addition of Ni and Bi to monometallic Pd improves the EOR stability and by-product tolerance of all ternary Pd_85_Ni_10_Bi_5_/C catalysts compared to commercial Pd/C catalyst. However, the durability of catalysts prepared by our modified instant reduction approach was not enhanced when compared to Pd_x_Ni_y_Bi_z_/C catalysts synthesized via the common instant reduction approach, suggesting there is room for further optimization of this process. Ongoing research is focused on advanced catalyst support materials such as reduced graphene oxide and N-doped graphene that may lead to overall improvements EOR stability and durability. Nevertheless, our synthesis scheme points to a new strategy for optimizing the structure, homogeneity, and overall performance of ternary PdNiBi catalysts on carbon supports.

## Electronic supplementary material

ESM1 (DOCX 11.5 mb)
