# Supplementary material for: Alkaline Ethanol Oxidation Reaction on Carbon Supported Ternary PdNiBi Nanocatalyst using Modified Instant Reduction Synthesis Method
Source: Electrocatalysis (N Y). 2020 Jan 3;11(2):203–14. doi: 10.1007/s12678-019-00577-8 (PMC7683445; doi:10.1007/s12678-019-00577-8)
Supplement: Supplementary file 1 — (DOCX 11.5 mb) [file 12678_2019_577_MOESM1_ESM.docx]

**SUPPLEMENTARY MATERIAL**

Alkaline Ethanol Oxidation Reaction on Carbon Supported Ternary PdNiBi Nanocatalyst using Modified Instant Reduction Synthesis Method

Bernd Cermenek^a,*^ (ORCID: [0000-0002-5133-7506](https://orcid.org/0000-0002-5133-7506)), Boštjan Genorio^b^ (ORCID: [0000-0002-0714-3472](https://orcid.org/0000-0002-0714-3472)), Thomas Winter^a^, Sigrid Wolf^a^, Justin G. Connell^c^, Michaela Roschger^a^, Ilse Letofsky-Papst^d^, Norbert Kienzl^e^, Brigitte Bitschnau^f^ (ORCID: [0000-0002-7669-2643](https://orcid.org/0000-0002-7669-2643)), Viktor Hacker ^a,*^ (ORCID: [0000-0001-5956-7579](https://orcid.org/0000-0001-5956-7579))

^a^Institute of Chemical Engineering and Environmental Technology, Fuel Cell Systems Group, Graz University of Technology, NAWI Graz, Inffeldgasse 25/C, 8010 Graz, Austria

^b^Faculty of Chemistry and Chemical Technology, University of Ljubljana, Večna pot 113, 1000 Ljubljana, Slovenia

^c^Materials Science Division, Argonne National Laboratory, 9700 South Cass Avenue, Lemont, Illinois 60439, USA

^d^Institute for Electron Microscopy and Nanoanalysis and Center for Electron Microscopy, Graz University of Technology, NAWI Graz, Steyrergasse 17, 8010 Graz, Austria

^e^Bioenergy 2020+ GmbH, Inffeldgasse 21/B, 8010 Graz, Austria

^f^Institute of Physical and Theoretical Chemistry, Graz University of Technology, Stremayrgasse 9, 8010 Graz, Austria

***Corresponding authors:**

Bernd Cermenek, Viktor Hacker, email: [bernd.cermenek@tugraz.at](mailto:bernd.cermenek@tugraz.at), [viktor.hacker@tugraz.at](mailto:viktor.hacker@tugraz.at)

Tel: +43 316 873 8781; Institute of Chemical Engineering and Environmental Technology, Fuel Cell Systems Group, Graz University of Technology, NAWI Graz, Inffeldgasse 25/C, 8010 Graz, Austria.

Table S1 ICP-OES data of the commercial Pd/C catalyst and the carbon supported Pd_85_Ni_10_B_5_ catalysts using the instant method with different modifications

| Catalysts |  | Pd  (g/kg) | Ni  (g/kg) | Bi  (g/kg) | Pd  (mol/kg) | Ni  (mol/kg) | Bi  (mol/kg) | Sum (mol/kg) | Pd  (at.%) | Ni  (at.%) | Bi  (at.%) |
| --- | --- | --- | --- | --- | --- | --- | --- | --- | --- | --- | --- |
| Pd/C comm. | Average | 406 | - | - | 3.82 | - | - | 3.82 | 100 | - | - |
| Pd_85_Ni_10_Bi_5_/C^(I)^ | Average | 173 | 11.9 | 21.3 | 1.63 | 0.20 | 0.10 | 1.93 | 84.2 | 10.5 | 5.3 |
| Pd_85_Ni_10_Bi_5_/C^(II)^ | Average | 335 | 21.3 | 41.6 | 3.15 | 0.36 | 0.20 | 3.71 | 84.9 | 9.8 | 5.4 |
| Pd_85_Ni_10_Bi_5_/C^(III)^ | Average | 339 | 21.2 | 36.8 | 3.19 | 0.36 | 0.18 | 3.72 | 85.6 | 9.7 | 4.7 |
| Atomic weight (AMU): Pd = 106.42; Ni = 58.69; Bi = 208.98 | | | | | | | | | | | |

| 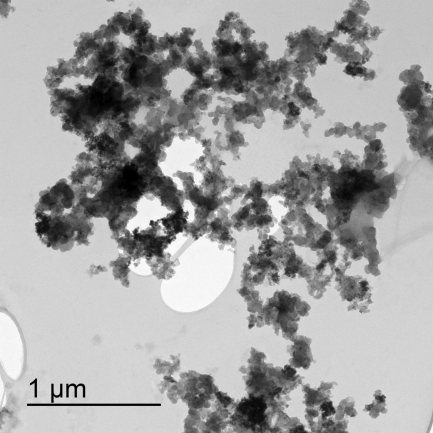 | 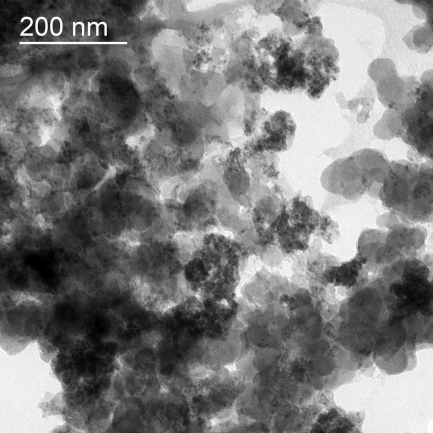 | 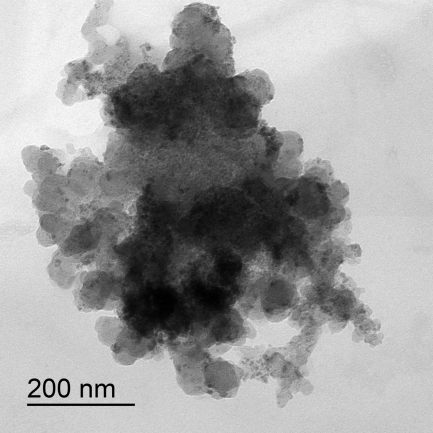 |
| --- | --- | --- |
| 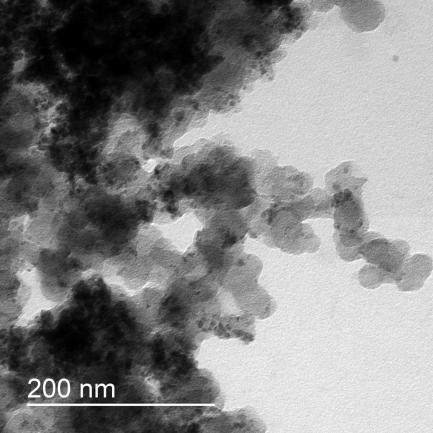 | 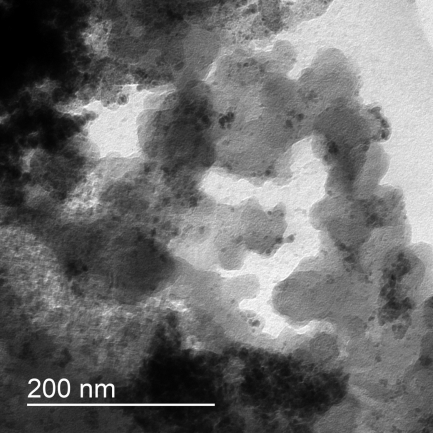 | 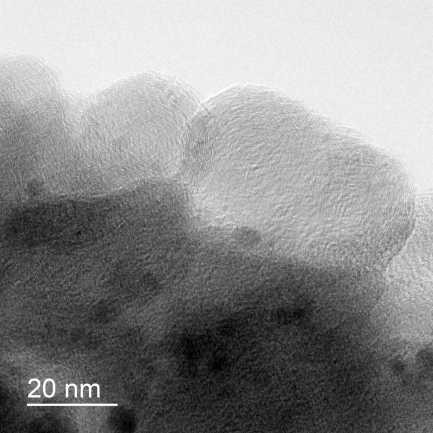 |
| 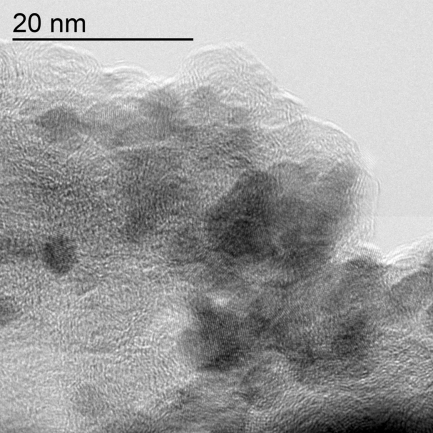 | 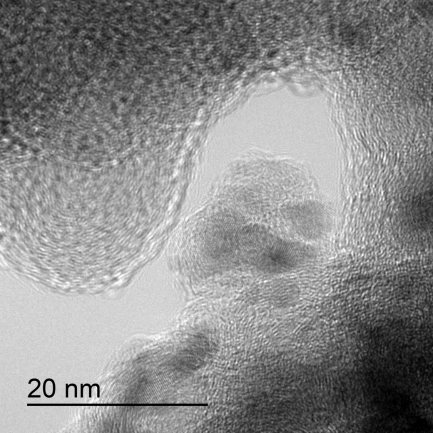 | 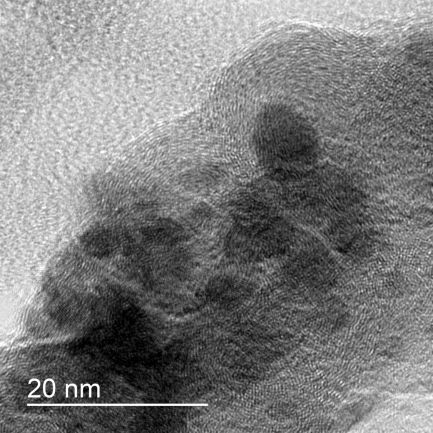 |

Fig. S1 TEM micrographs with low and high magnifications of the Pd_85_Ni_10_Bi_5_/C^(I)^ catalyst

| **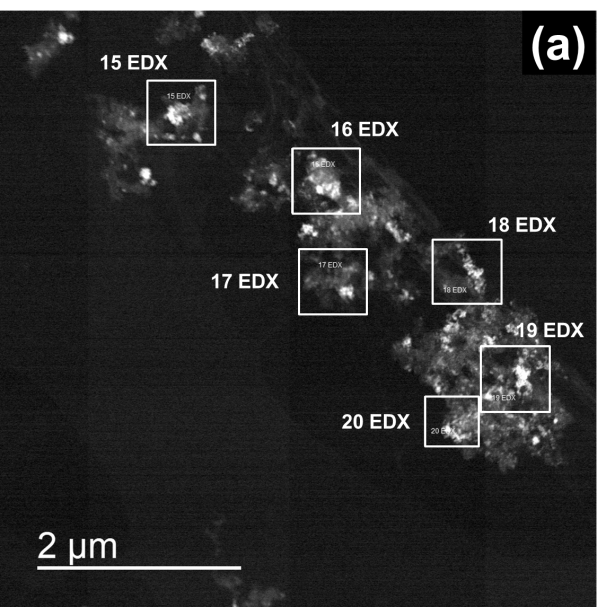** | |
| --- | --- |
| **** | **** |
| **** | **** |
| **** | **** |

Fig. S2 STEM-HAADF micrograph (a) and the corresponding EDX spectra (b)-(g) of the Pd_85_Ni_10_Bi_5_/C^(I)^
catalyst

Table S2 TEM-EDX results of the Pd_85_Ni_10_Bi_5_/C^(I)^ catalyst

| EDX areas | 3 elements | Net  Counts | k-factor  (Si) | rel.  (wt.%) | wt.%  (norm.) | at.% | rel.  (at.%) |
| --- | --- | --- | --- | --- | --- | --- | --- |
| 15 | Pd-L | 1203 | 2.651 | 1.000 | 82.85 | 82.29 | 1.000 |
|  | Ni-K | 176 | 1.525 | 0.084 | 6.97 | 12.56 | 0.153 |
|  | Bi-L | 61 | 6.425 | 0.123 | 10.18 | 5.15 | 0.063 |
| 16 | Pd-L | 1638 | 2.651 | 1.000 | 87.43 | 87.05 | 1.000 |
|  | Ni-K | 165 | 1.525 | 0.058 | 5.07 | 9.15 | 0.105 |
|  | Bi-L | 58 | 6.425 | 0.086 | 7.50 | 3.80 | 0.044 |
| 17 | Pd-L | 2308 | 2.651 | 1.000 | 82.11 | 83.59 | 1.000 |
|  | Ni-K | 263 | 1.525 | 0.066 | 5.38 | 9.93 | 0.119 |
|  | Bi-L | 145 | 6.425 | 0.152 | 12.50 | 6.48 | 0.078 |
| 18 | Pd-L | 2403 | 2.651 | 1.000 | 82.61 | 84.24 | 1.000 |
|  | Ni-K | 256 | 1.525 | 0.061 | 5.06 | 9.36 | 0.111 |
|  | Bi-L | 148 | 6.425 | 0.149 | 12.33 | 6.40 | 0.076 |
| 19 | Pd-L | 4096 | 2.651 | 1.000 | 80.96 | 82.83 | 1.000 |
|  | Ni-K | 478 | 1.525 | 0.067 | 5.43 | 10.08 | 0.122 |
|  | Bi-L | 284 | 6.425 | 0.168 | 13.60 | 7.09 | 0.086 |
| 20 | Pd-L | 2352 | 2.651 | 1.000 | 81.36 | 82.75 | 1.000 |
|  | Ni-K | 288 | 1.525 | 0.070 | 5.73 | 10.57 | 0.128 |
|  | Bi-L | 154 | 6.425 | 0.159 | 12.91 | 6.69 | 0.081 |

| 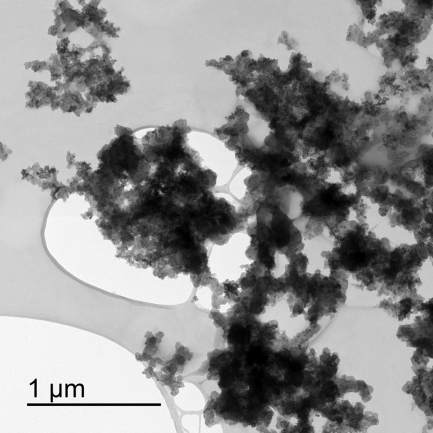 | 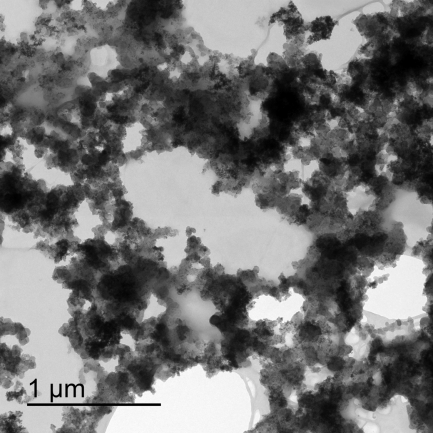 | 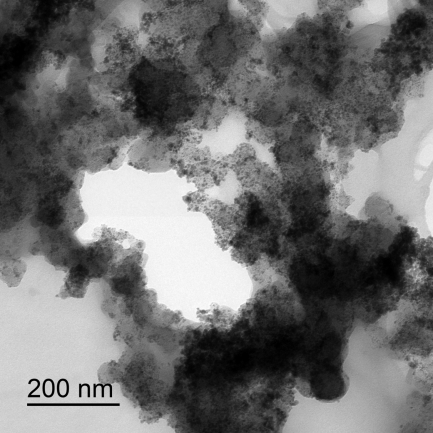 |
| --- | --- | --- |
| 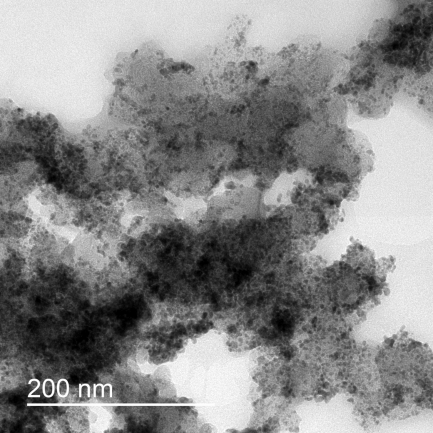 | 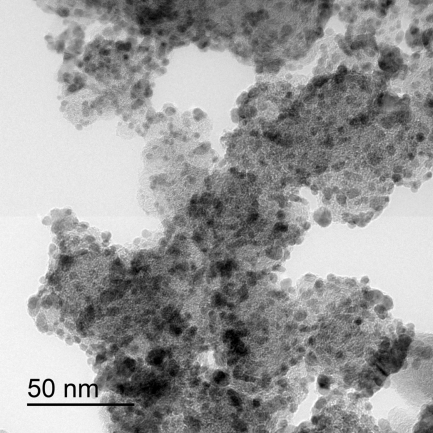 | 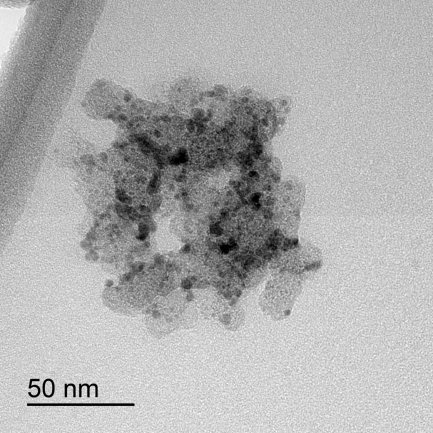 |
| 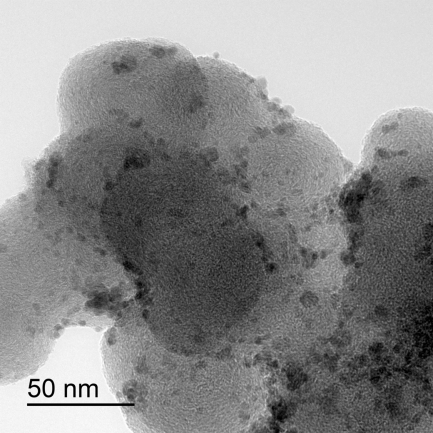 | 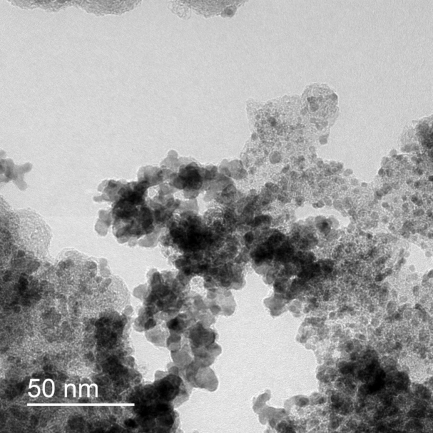 | 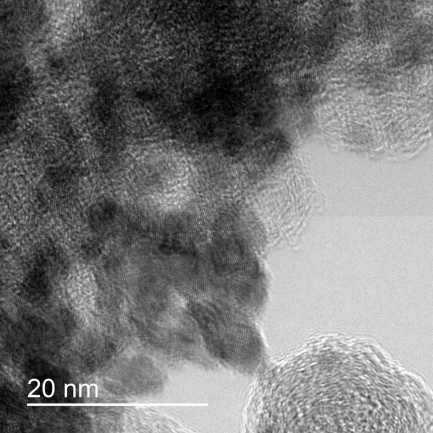 |
| 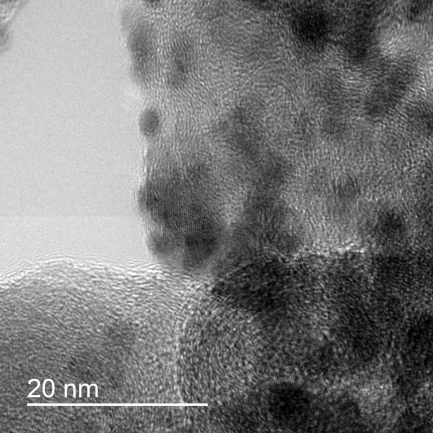 | 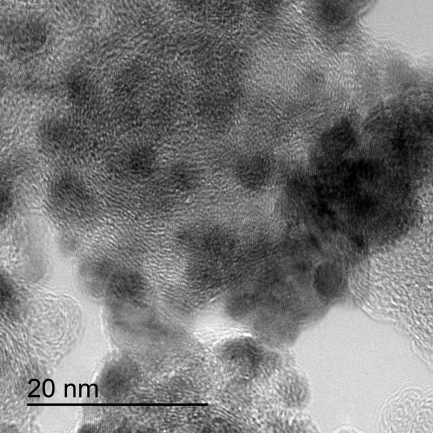 | 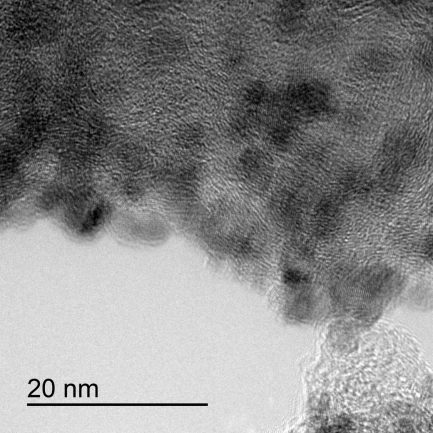 |

Fig. S3 TEM micrographs with low and high magnifications of the Pd_85_Ni_10_Bi_5_/C^(II)^ catalyst

| **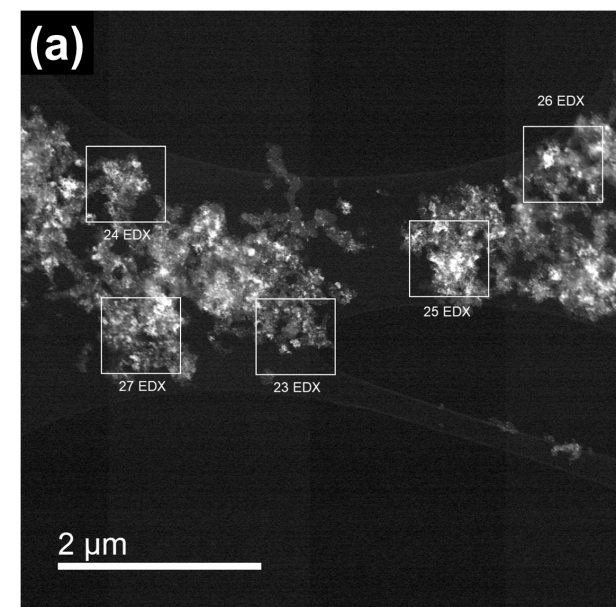** | **** |
| --- | --- |
| **** | **** |
| **** | **** |

Fig. S4 STEM-HAADF micrograph (a) and the corresponding EDX spectra (b)-(f) of the Pd_85_Ni_10_Bi_5_/C^(II)^ catalyst

Table S3 TEM-EDX results of the Pd_85_Ni_10_Bi_5_/C^(II)^ catalyst

| EDX areas | 3 elements | Net  counts | k-factor  (Si) | rel.  (wt.%) | wt.%  (norm.) | at.% | rel.  (at.%) |
| --- | --- | --- | --- | --- | --- | --- | --- |
| 23 | Pd-L | 3344 | 2.651 | 1.000 | 84.48 | 85.09 | 1.000 |
|  | Ni-K | 364 | 1.525 | 0.063 | 5.29 | 9.66 | 0.114 |
|  | Bi-L | 167 | 6.425 | 0.121 | 10.23 | 5.24 | 0.062 |
| 24 | Pd-L | 29847 | 2.651 | 1.000 | 82.66 | 84.09 | 1.000 |
|  | Ni-K | 3280 | 1.525 | 0.063 | 5.23 | 9.64 | 0.115 |
|  | Bi-L | 1804 | 6.425 | 0.146 | 12.11 | 6.27 | 0.075 |
| 25 | Pd-L | 3265 | 2.651 | 1.000 | 82.59 | 84.62 | 1.000 |
|  | Ni-K | 324 | 1.525 | 0.057 | 4.71 | 8.76 | 0.103 |
|  | Bi-L | 207 | 6.425 | 0.154 | 12.69 | 6.62 | 0.078 |
| 26 | Pd-L | 3107 | 2.651 | 1.000 | 82.04 | 84.07 | 1.000 |
|  | Ni-K | 323 | 1.525 | 0.060 | 4.91 | 9.12 | 0.108 |
|  | Bi-L | 204 | 6.425 | 0.159 | 13.05 | 6.81 | 0.081 |
| 27 | Pd-L | 2196 | 2.651 | 1.000 | 83.67 | 84.77 | 1.000 |
|  | Ni-K | 235 | 1.525 | 0.062 | 5.15% | 9.46 | 0.112 |
|  | Bi-L | 121 | 6.425 | 0.134 | 11.17% | 5.76 | 0.068 |

| 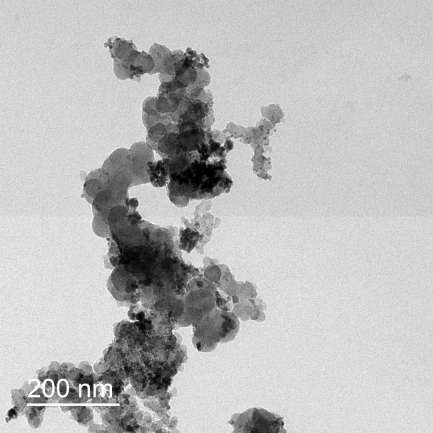 | 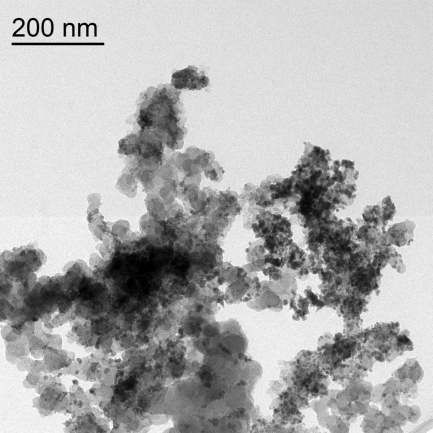 | 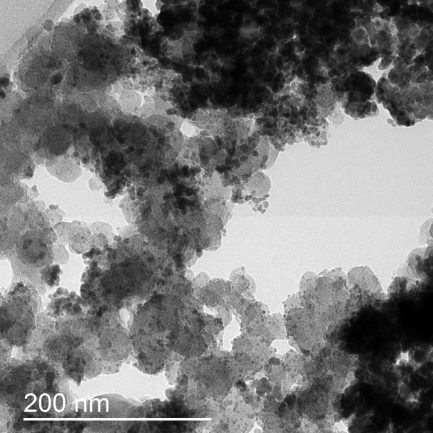 |
| --- | --- | --- |
| 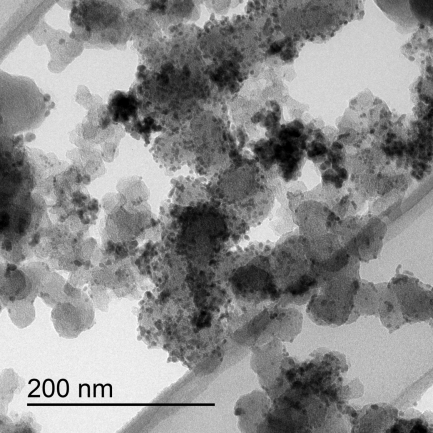 | 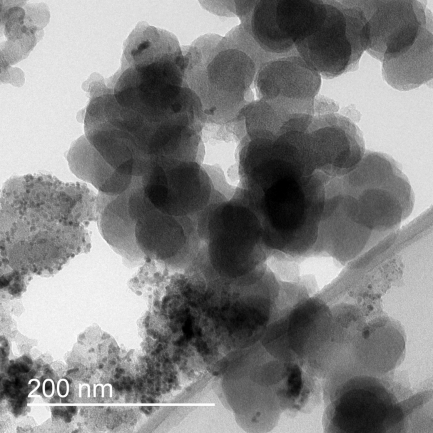 | 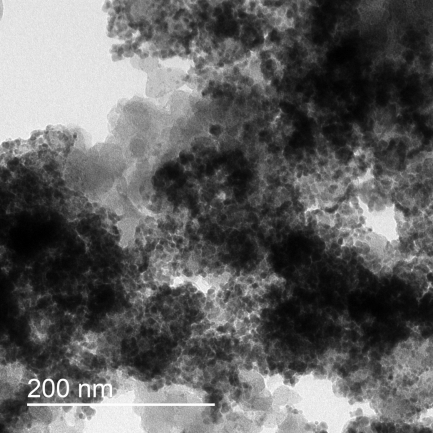 |
| 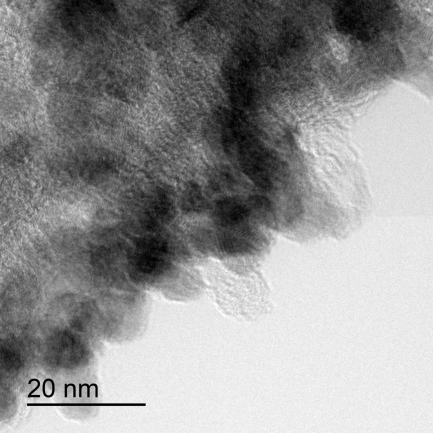 | 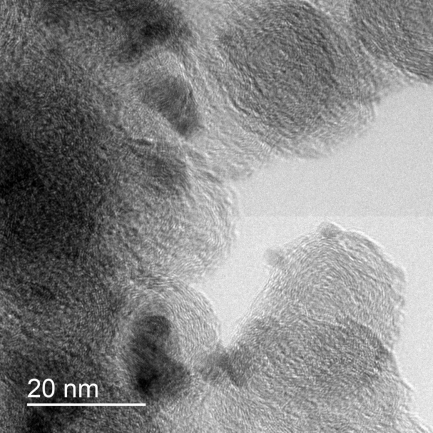 | 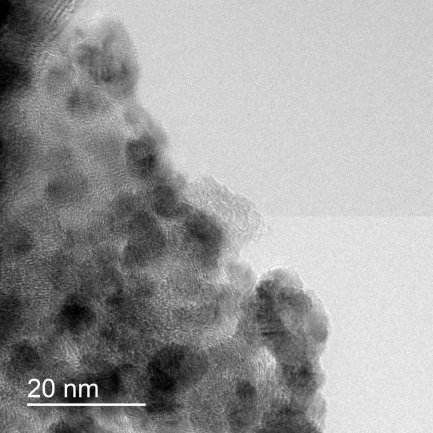 |
| 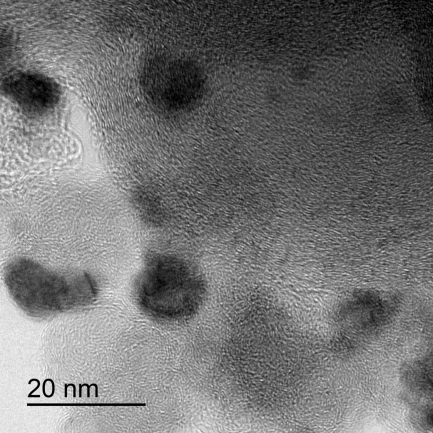 | 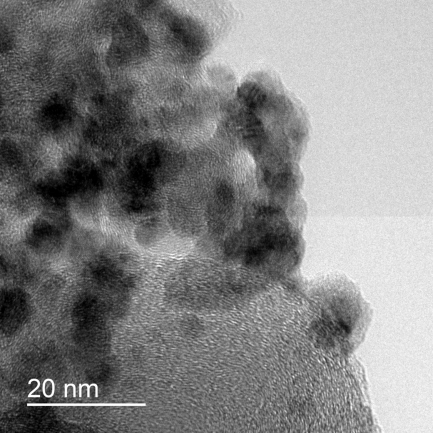 | 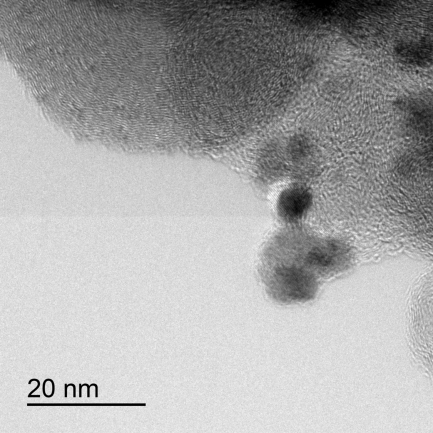 |

Fig. S5 TEM micrographs with low and high magnifications of the Pd_85_Ni_10_Bi_5_/C^(III)^ catalyst

| **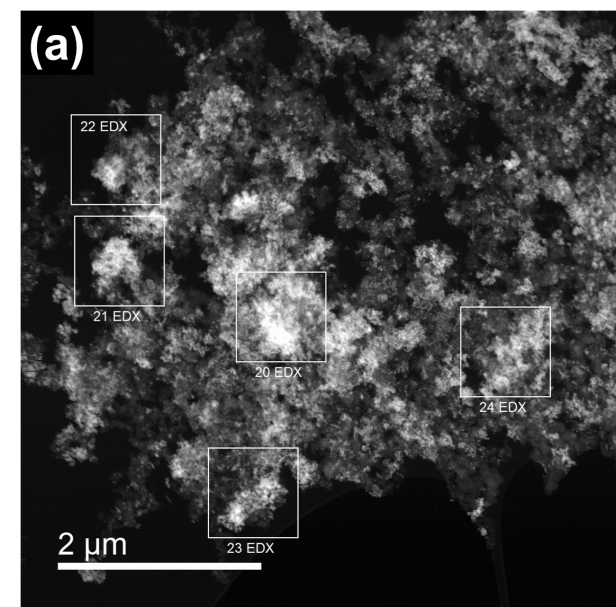** | **** |
| --- | --- |
| **** | **** |
| **** | **** |

Fig. S6 STEM-HAADF micrograph (a) and the corresponding EDX spectra (b)-(f) of the Pd_85_Ni_10_Bi_5_/C^(III)^ catalyst

Table S4 TEM-EDX results of the Pd_85_Ni_10_Bi_5_/C^(III)^ catalyst

| EDX areas | 3 elements | Net  counts | k-factor  (Si) | rel.  (wt.%) | wt.%  (norm.) | at.% | rel.  (at.%) |
| --- | --- | --- | --- | --- | --- | --- | --- |
| 20 | Pd-L | 6631 | 2.651 | 1.000 | 85.21 | 86.29 | 1.000 |
|  | Ni-K | 623 | 1.525 | 0.054 | 4.61 | 8.46 | 0.098 |
|  | Bi-L | 327 | 6.425 | 0.120 | 10.18 | 5.25 | 0.061 |
| 21 | Pd-L | 4744 | 2.651 | 1.000 | 85.19 | 85.43 | 1.000 |
|  | Ni-K | 519 | 1.525 | 0.063 | 5.36 | 9.75 | 0.114 |
|  | Bi-L | 217 | 6.425 | 0.111 | 9.44 | 4.82 | 0.056 |
| 22 | Pd-L | 4663 | 2.651 | 1.000 | 83.38 | 84.38 | 1.000 |
|  | Ni-K | 520 | 1.525 | 0.064 | 5.35 | 9.81 | 0.116 |
|  | Bi-L | 260 | 6.425 | 0.135 | 11.27 | 5.81 | 0.069 |
| 23 | Pd-L | 5152 | 2.651 | 1.000 | 81.66 | 81.76 | 1.000 |
|  | Ni-K | 747 | 1.525 | 0.083 | 6.81 | 12.36 | 0.151 |
|  | Bi-L | 300 | 6.425 | 0.141 | 11.52 | 5.88 | 0.072 |
| 24 | Pd-L | 3359 | 2.651 | 1.000 | 78.84 | 79.31 | 1.000 |
|  | Ni-K | 556 | 1.525 | 0.095 | 7.51 | 13.69 | 0.173 |
|  | Bi-L | 240 | 6.425 | 0.173 | 13.65 | 6.99 | 0.088 |

Table S5 XRD data (Rietveld refinement) of the commercial Pd/C catalyst

| Catalyst | Peak  No. | Peak Pos.  (° 2θ) | *d*-spacing  (Å) | Rel. Int.  (%) | FWHM  (° 2θ) | h | k | l | Assignment |
| --- | --- | --- | --- | --- | --- | --- | --- | --- | --- |
| Pd/C comm | 1 | 24.756 | 3.593 | 1.3 | 2.414 | 0 | 0 | 2 | Graphite(2H)_187640-ICSD |
|  | 2 | 27.303 | 3.264 | 0.1 | 1.970 | 1 | 1 | 1 | NaCl_655785-ICSD |
|  | 3 | 31.640 | 2.826 | 1.4 | 1.970 | 0 | 0 | 2 | NaCl_655785-ICSD |
|  | 4 | 39.246 | 2.294 | 0.1 | 2.414 | 0 | 1 | 0 | Graphite(2H)_187640-ICSD |
|  | 5 | 40.057 | 2.249 | 100.0 | 1.143 | 1 | 1 | 1 | Pd_64922-ICSD |
|  | 6 | 41.291 | 2.185 | 0.2 | 2.414 | 0 | 1 | 1 | Graphite(2H)_187640-ICSD |
|  | 7 | 45.385 | 1.997 | 0.9 | 1.970 | 0 | 2 | 2 | NaCl_655785-ICSD |
|  | 8 | 46.602 | 1.947 | 28.1 | 1.159 | 0 | 0 | 2 | Pd_64922-ICSD |
|  | 9 | 46.986 | 1.932 | 0.1 | 2.414 | 0 | 1 | 2 | Graphite(2H)_187640-ICSD |
|  | 10 | 50.847 | 1.794 | 0.1 | 2.414 | 0 | 0 | 4 | Graphite(2H)_187640-ICSD |
|  | 11 | 55.472 | 1.655 | 0.1 | 2.414 | 0 | 1 | 3 | Graphite(2H)_187640-ICSD |
|  | 12 | 56.410 | 1.630 | 0.3 | 1.970 | 2 | 2 | 2 | NaCl_655785-ICSD |
|  | 13 | 66.164 | 1.411 | 0.1 | 1.970 | 0 | 0 | 4 | NaCl_655785-ICSD |
|  | 14 | 68.067 | 1.376 | 12.5 | 1.238 | 0 | 2 | 2 | Pd_64922-ICSD |
|  | 15 | 71.202 | 1.323 | 0.1 | 2.414 | 1 | 1 | 0 | Graphite(2H)_187640-ICSD |
|  | 16 | 75.230 | 1.262 | 0.3 | 1.970 | 0 | 2 | 4 | NaCl_655785-ICSD |
|  | 17 | 76.709 | 1.241 | 0.1 | 2.414 | 1 | 1 | 2 | Graphite(2H)_187640-ICSD |
|  | 18 | 82.052 | 1.174 | 10.7 | 1.321 | 1 | 1 | 3 | Pd_64922-ICSD |
|  | 19 | 83.930 | 1.152 | 0.3 | 1.970 | 2 | 2 | 4 | NaCl_655785-ICSD |
|  | 20 | 86.569 | 1.124 | 3.2 | 1.355 | 2 | 2 | 2 | Pd_64922-ICSD |
|  | 21 | 92.705 | 1.065 | 0.1 | 2.414 | 1 | 1 | 4 | Graphite(2H)_187640-ICSD |

Table S6 XRD data (Rietveld refinement) of the Pd_85_Ni_10_Bi_5_/C^(I)^ catalyst

| Catalyst | Peak No. | Peak Pos.  (° 2θ) | *d*-spacing  (Å) | Rel. Int.  (%) | FWHM  (° 2θ) | h | k | l | Assignment |
| --- | --- | --- | --- | --- | --- | --- | --- | --- | --- |
| Pd_85_Ni_10_Bi_5_/C^(I)^ | 1 | 25.038 | 3.554 | 8.2 | 3.162 | 0 | 0 | 2 | Graphite(2H)_187640-ICSD |
|  | 2 | 39.821 | 2.262 | 100.0 | 2.550 | 1 | 1 | 1 | Pd_64922-ICSD |
|  | 3 | 42.452 | 2.128 | 0.2 | 3.162 | 0 | 1 | 0 | Graphite(2H)_187640-ICSD |
|  | 4 | 44.366 | 2.040 | 1.2 | 3.162 | 0 | 1 | 1 | Graphite(2H)_187640-ICSD |
|  | 5 | 46.243 | 1.962 | 23.2 | 2.648 | 0 | 0 | 2 | Pd_64922-ICSD |
|  | 6 | 49.755 | 1.831 | 0.3 | 3.162 | 0 | 1 | 2 | Graphite(2H)_187640-ICSD |
|  | 7 | 50.943 | 1.791 | 0.6 | 3.162 | 0 | 0 | 4 | Graphite(2H)_187640-ICSD |
|  | 8 | 57.893 | 1.592 | 0.4 | 3.162 | 0 | 1 | 3 | Graphite(2H)_187640-ICSD |
|  | 9 | 67.256 | 1.391 | 11.7 | 3.095 | 0 | 2 | 2 | Pd_64922-ICSD |
|  | 10 | 68.200 | 1.374 | 0.1 | 3.162 | 0 | 1 | 4 | Graphite(2H)_187640-ICSD |
|  | 11 | 77.267 | 1.234 | 0.4 | 3.162 | 1 | 1 | 0 | Graphite(2H)_187640-ICSD |
|  | 12 | 80.055 | 1.198 | 0.1 | 3.162 | 0 | 0 | 6 | Graphite(2H)_187640-ICSD |
|  | 13 | 80.458 | 1.193 | 0.1 | 3.162 | 0 | 1 | 5 | Graphite(2H)_187640-ICSD |
|  | 14 | 80.883 | 1.188 | 10.5 | 3.520 | 1 | 1 | 3 | Pd_64922-ICSD |
|  | 15 | 82.582 | 1.167 | 0.7 | 3.162 | 1 | 1 | 2 | Graphite(2H)_187640-ICSD |
|  | 16 | 85.269 | 1.137 | 3.5 | 3.686 | 2 | 2 | 2 | Pd_64922-ICSD |
|  | 17 | 92.147 | 1.070 | 0.0 | 3.162 | 0 | 2 | 0 | Graphite(2H)_187640-ICSD |
|  | 18 | 93.455 | 1.058 | 0.1 | 3.162 | 0 | 2 | 1 | Graphite(2H)_187640-ICSD |
|  | 19 | 94.881 | 1.046 | 0.0 | 3.162 | 0 | 1 | 6 | Graphite(2H)_187640-ICSD |
|  | 20 | 97.390 | 1.025 | 0.0 | 3.162 | 0 | 2 | 2 | Graphite(2H)_187640-ICSD |
|  | 21 | 98.307 | 1.018 | 0.5 | 3.162 | 1 | 1 | 4 | Graphite(2H)_187640-ICSD |
|  | 22 | 25.038 | 3.554 | 8.2 | 3.162 | 0 | 0 | 2 | Graphite(2H)_187640-ICSD |

Table S7 XRD data (Rietveld refinement) of the Pd_85_Ni_10_Bi_5_/C^(II)^ catalyst

| Catalyst | Peak No. | Pos.  (° 2θ) | *d*-spacing  (Å) | Rel. Int.  (%) | FWHM  (° 2θ) | h | k | l | Assignment |
| --- | --- | --- | --- | --- | --- | --- | --- | --- | --- |
| Pd_85_Ni_10_Bi_5_/C^(II)^ | 1 | 24.815 | 3.585 | 6.5 | 2.336 | 0 | 0 | 2 | Graphite(2H)_187640-ICSD |
|  | 2 | 38.458 | 2.339 | 0.3 | 2.336 | 0 | 1 | 0 | Graphite(2H)_187640-ICSD |
|  | 3 | 39.570 | 2.276 | 100.0 | 2.660 | 1 | 1 | 1 | Pd_64922-ICSD |
|  | 4 | 40.519 | 2.225 | 1.3 | 2.336 | 0 | 1 | 1 | Graphite(2H)_187640-ICSD |
|  | 5 | 45.987 | 1.972 | 9.0 | 2.650 | 0 | 0 | 2 | Pd_64922-ICSD |
|  | 6 | 46.244 | 1.962 | 0.3 | 2.336 | 0 | 1 | 2 | Graphite(2H)_187640-ICSD |
|  | 7 | 50.720 | 1.798 | 0.5 | 2.336 | 0 | 0 | 4 | Graphite(2H)_187640-ICSD |
|  | 8 | 54.737 | 1.676 | 0.4 | 2.336 | 0 | 1 | 3 | Graphite(2H)_187640-ICSD |
|  | 9 | 65.324 | 1.427 | 0.1 | 2.336 | 0 | 1 | 4 | Graphite(2H)_187640-ICSD |
|  | 10 | 66.981 | 1.396 | 2.7 | 2.601 | 0 | 2 | 2 | Pd_64922-ICSD |
|  | 11 | 69.409 | 1.353 | 0.4 | 2.336 | 1 | 1 | 0 | Graphite(2H)_187640-ICSD |
|  | 12 | 74.906 | 1.267 | 0.7 | 2.336 | 1 | 1 | 2 | Graphite(2H)_187640-ICSD |
|  | 13 | 77.754 | 1.227 | 0.1 | 2.336 | 0 | 1 | 5 | Graphite(2H)_187640-ICSD |
|  | 14 | 79.831 | 1.200 | 0.1 | 2.336 | 0 | 0 | 6 | Graphite(2H)_187640-ICSD |
|  | 15 | 80.594 | 1.191 | 1.5 | 2.547 | 1 | 1 | 3 | Pd_64922-ICSD |
|  | 16 | 83.484 | 1.157 | 0.1 | 2.336 | 0 | 2 | 1 | Graphite(2H)_187640-ICSD |
|  | 17 | 84.975 | 1.140 | 0.6 | 2.523 | 2 | 2 | 2 | Pd_64922-ICSD |
|  | 18 | 87.417 | 1.115 | 0.0 | 2.336 | 0 | 2 | 2 | Graphite(2H)_187640-ICSD |
|  | 19 | 90.761 | 1.082 | 0.4 | 2.336 | 1 | 1 | 4 | Graphite(2H)_187640-ICSD |
|  | 20 | 92.221 | 1.069 | 0.0 | 2.336 | 0 | 1 | 6 | Graphite(2H)_187640-ICSD |
|  | 21 | 93.953 | 1.054 | 0.1 | 2.336 | 0 | 2 | 3 | Graphite(2H)_187640-ICSD |

Table S8 XRD data (Rietveld refinement) of the Pd_85_Ni_10_Bi_5_/C^(III)^ catalyst

| Catalyst | Peak No. | Pos.  (° 2θ) | *d*-spacing  (Å) | Rel. Int.  (%) | FWHM  (° 2θ) | h | k | l | Assignment |
| --- | --- | --- | --- | --- | --- | --- | --- | --- | --- |
| Pd_85_Ni_10_Bi_5_/C^(III)^ | 1 | 25.273 | 3.521 | 4.4 | 2.441 | 0 | 0 | 2 | Graphite(2H)_187640-ICSD |
|  | 2 | 39.227 | 2.295 | 100.0 | 2.269 | 1 | 1 | 1 | Pd_64922-ICSD |
|  | 3 | 42.086 | 2.145 | 0.1 | 2.441 | 0 | 1 | 0 | Graphite(2H)_187640-ICSD |
|  | 4 | 44.033 | 2.055 | 0.7 | 2.441 | 0 | 1 | 1 | Graphite(2H)_187640-ICSD |
|  | 5 | 45.518 | 1.991 | 22.8 | 2.258 | 0 | 0 | 2 | Pd_64922-ICSD |
|  | 6 | 49.502 | 1.840 | 0.1 | 2.441 | 0 | 1 | 2 | Graphite(2H)_187640-ICSD |
|  | 7 | 51.290 | 1.780 | 0.3 | 2.441 | 0 | 0 | 4 | Graphite(2H)_187640-ICSD |
|  | 8 | 57.740 | 1.595 | 0.2 | 2.441 | 0 | 1 | 3 | Graphite(2H)_187640-ICSD |
|  | 9 | 66.049 | 1.413 | 8.9 | 2.204 | 0 | 2 | 2 | Pd_64922-ICSD |
|  | 10 | 68.150 | 1.375 | 0.0 | 2.441 | 0 | 1 | 4 | Graphite(2H)_187640-ICSD |
|  | 11 | 76.368 | 1.246 | 0.2 | 2.441 | 1 | 1 | 0 | Graphite(2H)_187640-ICSD |
|  | 12 | 79.302 | 1.207 | 6.6 | 2.144 | 1 | 1 | 3 | Pd_64922-ICSD |
|  | 13 | 80.511 | 1.192 | 0.1 | 2.441 | 0 | 1 | 5 | Graphite(2H)_187640-ICSD |
|  | 14 | 80.563 | 1.191 | 0.1 | 2.441 | 0 | 0 | 6 | Graphite(2H)_187640-ICSD |
|  | 15 | 81.742 | 1.177 | 0.4 | 2.441 | 1 | 1 | 2 | Graphite(2H)_187640-ICSD |
|  | 16 | 83.552 | 1.156 | 2.1 | 2.118 | 2 | 2 | 2 | Pd_64922-ICSD |
|  | 17 | 90.941 | 1.081 | 0.0 | 2.441 | 0 | 2 | 0 | Graphite(2H)_187640-ICSD |
|  | 18 | 92.258 | 1.069 | 0.1 | 2.441 | 0 | 2 | 1 | Graphite(2H)_187640-ICSD |
|  | 19 | 95.048 | 1.044 | 0.0 | 2.441 | 0 | 1 | 6 | Graphite(2H)_187640-ICSD |
|  | 20 | 95.997 | 1.037 | 0.1 | 2.251 | 0 | 2 | 3 | Graphite(2H)_187640-ICSD |
|  | 21 | 96.216 | 1.035 | 0.0 | 2.441 | 0 | 2 | 2 | Graphite(2H)_187640-ICSD |
|  | 22 | 97.592 | 1.024 | 0.3 | 2.441 | 1 | 1 | 4 | Graphite(2H)_187640-ICSD |

Table S9 XRD analysis of the commercial Pd/C catalyst - average crystallite size estimation according to Scherrer. assuming spherical crystallites with cubic crystal system at five diffraction peaks (peak pos.) including the determination of half-width (B_obs_) and lattice parameter by profile fit and Rietveld refinement, respectively

| Catalyst | Peak No | B_obs._^a^  (° 2θ) | B_std._^b^  (° 2θ) | Peak pos.^c^  (° 2θ) | B_struct._^d^  (° 2θ) | Crystallite size (Å) | Crystallite size (nm) |
| --- | --- | --- | --- | --- | --- | --- | --- |
| Pd/C comm. | 1 | 1.143 | 0.046 | 40.057 | 1.054 | 86 | 9 |
|  | 2 | 1.159 | 0.048 | 46.602 | 1.342 | 87 | 9 |
|  | 3 | 1.238 | 0.055 | 68.067 | 1.265 | 90 | 9 |
|  | 4 | 1.321 | 0.060 | 82.052 | 1.290 | 93 | 9 |
|  | 5 | 1.355 | 0.064 | 86.569 | 1.290 | 94 | 9 |
| Lattice parameter: a = 3.890 Å | | | | | | | |

^a^B_obs._: half width (full width half maximum FWHM. peak width) - obs. = observed; ^b^B_std_: device-related width. Derived from LaB_6_ - std. = standard; ^c^pos.: position; ^d^B_struct._: Difference between B_obs._ and B_std_ - struct. = structural.

Table S10 XRD analysis of the Pd_85_Ni_10_Bi_5_/C^(I)^ catalyst - average crystallite size estimation according to Scherrer. assuming spherical crystallites with cubic crystal system at five diffraction peaks (peak pos.) including the determination of half-width (B_obs_) and lattice parameter by profile fit and Rietveld refinement, respectively

| Catalyst | Peak No | B_obs._^a^  (° 2θ) | B_std._^b^  (° 2θ) | Peak pos.^c^  (° 2θ) | B_struct._^d^  (° 2θ) | Crystallite size (Å) | Crystallite size (nm) |
| --- | --- | --- | --- | --- | --- | --- | --- |
| Pd_85_Ni_10_Bi_5_/C^(I)^ | 1 | 2.550 | 0.046 | 39.821 | 2.504 | 38 | 4 |
|  | 2 | 2.648 | 0.048 | 46.243 | 2.600 | 37 | 4 |
|  | 3 | 3.095 | 0.055 | 67.256 | 3.040 | 35 | 3 |
|  | 4 | 3.520 | 0.060 | 80.883 | 3.460 | 34 | 3 |
|  | 5 | 3.686 | 0.064 | 85.269 | 3.622 | 33 | 3 |
| Lattice parameter: a = 3.953 Å | | | | | | | |

^a^B_obs._: half width (full width half maximum FWHM. peak width) - obs. = observed; ^b^B_std_: device-related width. Derived from LaB_6_ - std. = standard; ^c^pos.: position; ^d^B_struct._: Difference between B_obs._ and B_std_ - struct. = structural.

Table S11 XRD analysis of the Pd_85_Ni_10_Bi_5_/C^(II)^ catalyst - average crystallite size estimation according to Scherrer assuming spherical crystallites with cubic crystal system at five diffraction peaks (peak pos.) including the determination of half-width (B_obs_) and lattice parameter by profile fit and Rietveld refinement, respectively

| Catalyst | Peak No | B_obs._^a^  (° 2θ) | B_std._^b^  (° 2θ) | Peak pos.^c^  (° 2θ) | B_struct._^d^  (° 2θ) | Crystallite size (Å) | Crystallite size (nm) |
| --- | --- | --- | --- | --- | --- | --- | --- |
| Pd_85_Ni_10_Bi_5_/C^(II)^ | 1 | 2.660 | 0.046 | 39.570 | 2.614 | 36 | 4 |
|  | 2 | 2.650 | 0.048 | 45.987 | 2.602 | 37 | 4 |
|  | 3 | 2.601 | 0.055 | 66.981 | 2.546 | 42 | 4 |
|  | 4 | 2.547 | 0.060 | 80.594 | 2.487 | 47 | 5 |
|  | 5 | 2.523 | 0.064 | 84.975 | 2.459 | 49 | 5 |
| Lattice parameter: a = 3.957 Å | | | | | | | |

^a^B_obs._: half width (full width half maximum FWHM. peak width) - obs. = observed; ^b^B_std_: device-related width. Derived from LaB_6_ - std. = standard; ^c^pos.: position; ^d^B_struct._: Difference between B_obs._ and B_std_ - struct. = structural.

Table S12 XRD analysis of the Pd_85_Ni_10_Bi_5_/C^(III)^ catalyst - average crystallite size estimation according to Scherrer. assuming spherical crystallites with cubic crystal system at five diffraction peaks (peak pos.) including the determination of half-width (B_obs_) and lattice parameter by profile fit and Rietveld refinement, respectively

| Catalyst | Peak No | B_obs._^a^  (° 2θ) | B_std._^b^  (° 2θ) | Peak pos.^c^  (° 2θ) | B_struct._^d^  (° 2θ) | Crystallite size (Å) | Crystallite size (nm) |
| --- | --- | --- | --- | --- | --- | --- | --- |
| Pd_85_Ni_10_Bi_5_/C^(III)^ | 1 | 2.269 | 0.046 | 39.227 | 2.223 | 42 | 4 |
|  | 2 | 2.258 | 0.048 | 45.518 | 2.210 | 43 | 4 |
|  | 3 | 2.204 | 0.055 | 66.049 | 2.149 | 49 | 5 |
|  | 4 | 2.144 | 0.060 | 79.302 | 2.084 | 55 | 5 |
|  | 5 | 2.118 | 0.064 | 83.552 | 2.054 | 58 | 6 |
| Lattice parameter: a = 4.024 Å | | | | | | | |

^a^B_obs._: half width (full width half maximum FWHM. peak width) - obs. = observed; ^b^B_std_: device-related width. Derived from LaB_6_ - std. = standard; ^c^pos.: position; ^d^B_struct._: Difference between B_obs._ and B_std_ - struct. = structural.


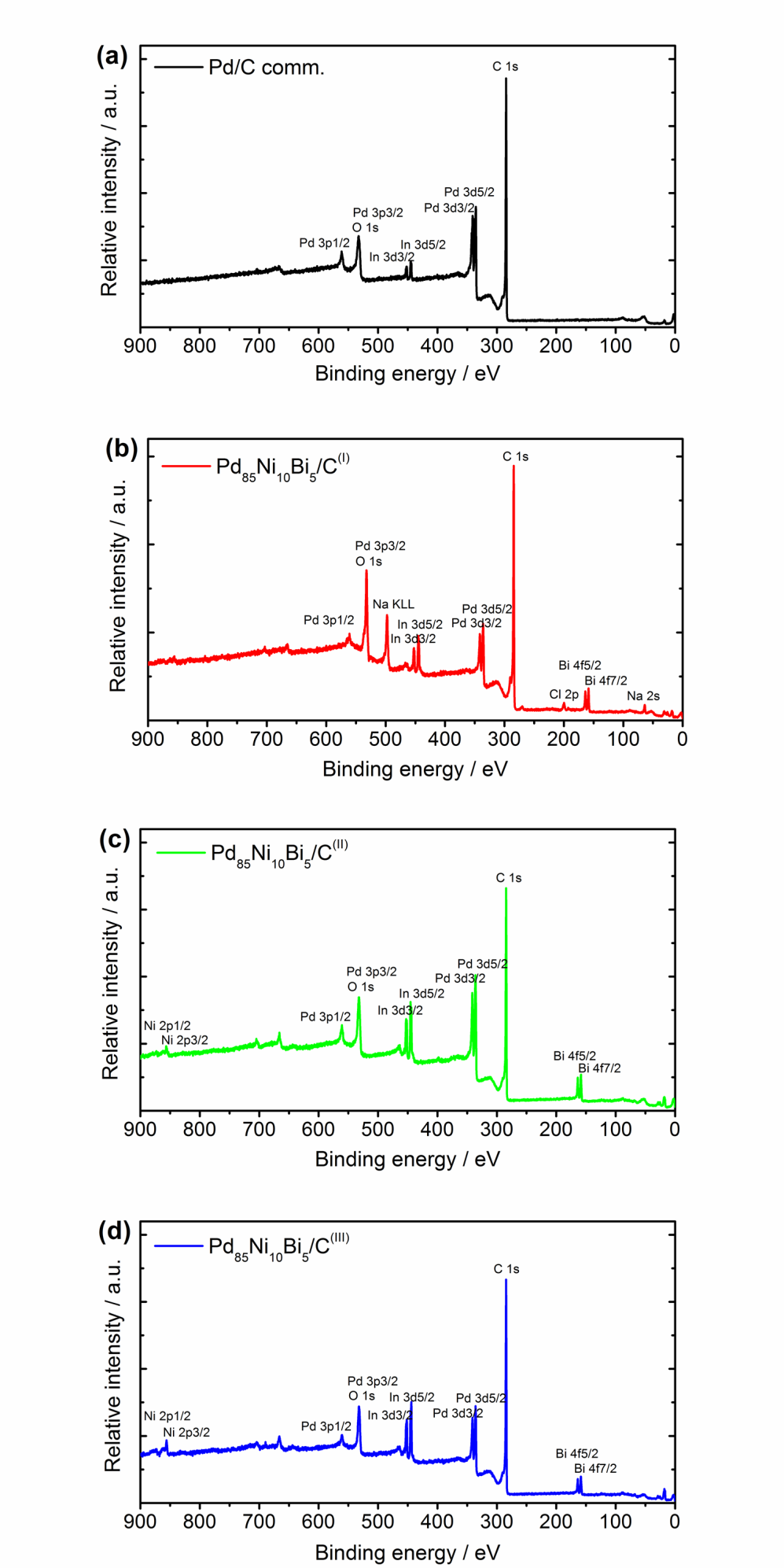


Fig. S7 Survey XPS spectra of the (a) Pd/C comm., (b) Pd_85_Ni_10_Bi_5_/C^(I)^, (c) Pd_85_Ni_10_Bi_5_/C^(II)^ and
(d) Pd_85_Ni_10_Bi_5_/C^(III)^ catalyst

Table S13 XPS data (measured) of the commercial Pd/C catalyst

| Catalyst | Peak Name | Peak Pos. (eV) | Raw Area | Area/(RSF*T*MFP)^a^ | At. Conc. (%)^b^ |
| --- | --- | --- | --- | --- | --- |
| Pd/C comm. | C 1s | 284.00 | 328619 | 328619 | 90.68 |
|  | Pd 3d | 335.00 | 385754 | 24049.5 | 6.64 |
|  | Bi 4f | 161.00 | -171.275 | -6.89792 | 0 |
|  | Ni 2p3/2 | 847.40 | -325.222 | -22.2603 | 0 |
|  | O 1s | 534.53 | 28471 | 9717.07 | 2.68 |

^a^RSF: relative sensitivity factor. T: transmission factor. MFP: mean free path; ^b^atomic concentration.

Table S14 XPS data (measured) of the Pd_85_Ni_10_Bi_5_/C^(I)^ catalyst

| Catalyst | Peak Name | Peak Pos. (eV) | Raw Area | Area/(RSF*T*MFP)^a^ | At. Conc. (%)^b^ |
| --- | --- | --- | --- | --- | --- |
| Pd_85_Ni_10_Bi_5_/C^(I)^ | C 1s | 284.00 | 270798 | 270798 | 89.65 |
|  | Pd 3d | 335.60 | 175750 | 10957 | 3.63 |
|  | Bi 4f | 158.00 | 46957.9 | 1891.18 | 0.63 |
|  | Ni 2p3/2 | 855.20 | 19169.4 | 1312.07 | 0.43 |
|  | O 1s | 530.90 | 50070.4 | 17088.9 | 5.66 |

^a^RSF: relative sensitivity factor. T: transmission factor. MFP: mean free path; ^b^atomic concentration.

Table S15 XPS data (measured) of the Pd_85_Ni_10_Bi_5_/C^(II)^ catalyst

| Catalyst | Peak Name | Peak Pos. (eV) | Raw Area | Area/(RSF*T*MFP)^a^ | At. Conc. (%)^b^ |
| --- | --- | --- | --- | --- | --- |
| Pd_85_Ni_10_Bi_5_/C^(II)^ | C 1s | 284.00 | 294509 | 294509 | 87.22 |
|  | Pd 3d | 335.60 | 366470 | 22847.3 | 6.77 |
|  | Bi 4f | 158.00 | 71298.9 | 2871.48 | 0.85 |
|  | Ni 2p3/2 | 856.20 | 32983.2 | 2257.57 | 0.67 |
|  | O 1s | 529.85 | 44430.2 | 15163.9 | 4.49 |

^a^RSF: relative sensitivity factor. T: transmission factor. MFP: mean free path; ^b^atomic concentration.

Table S16 XPS data (measured) of the Pd_85_Ni_10_Bi_5_/C^(III)^ catalyst

| Catalyst | Peak Name | Peak Pos. (eV) | Raw Area | Area/(RSF*T*MFP)^a^ | At. Conc. (%)^b^ |
| --- | --- | --- | --- | --- | --- |
| Pd_85_Ni_10_Bi_5_/C^(III)^ | C 1s | 283.95 | 292014 | 292014 | 90.41 |
|  | Pd 3d | 335.35 | 231567 | 14436.9 | 4.47 |
|  | Bi 4f | 158.15 | 51581.8 | 2077.4 | 0.64 |
|  | Ni 2p3/2 | 855.55 | 50236.4 | 3438.5 | 1.06 |
|  | O 1s | 530.07 | 32338.7 | 11037.1 | 3.42 |

^a^RSF: relative sensitivity factor. T: transmission factor. MFP: mean free path; ^b^atomic concentration.

Table S17 XPS results (fitted) of carbon supported Pd-based catalysts with the assignments of Pd 3d. Ni 2p and Bi 4f peaks including the determined metal contents obtained from measured data

| Catalyst | Pd 3d (eV) | Pd (at.%) | Assignment | Ni 2p (eV) | Ni (at.%) | Assignment | Bi 4f (eV) | Bi (at.%) | Assignment | Pd/ metal | Ni/ metal | Bi/ metal |
| --- | --- | --- | --- | --- | --- | --- | --- | --- | --- | --- | --- | --- |
| Pd/C comm. | 335.00  340.30 | 5.83 | Pd^0^ | - | - | - | - | - | - | **100** | **-** | **-** |
|  | 336.76  342.06 | 1.15 | PdO | - | - | - | - | - | - |  |  |  |
| Pd_85_Ni_10_Bi_5_/C^(I)^ | 335.15  340.45 | 3.17 | Pd^0^ | 855.74  854.97 | 0.20 | Ni(OH)_2_ | 158.10  163.40 | 0.49 | Bi^0^ | **77** | **9** | **13** |
|  | 336.53  341.83 | 0.88 | PdO | 857.74  860.53 | 0.02 | Ni(OH)_2_ | 159.50  164.80 | 0.16 | Bi_2_O_3_ |  |  |  |
|  |  |  |  | 861.53  866.66 | 0.16 | Ni(OH)_2_ |  |  |  |  |  |  |
| Pd_85_Ni_10_Bi_5_/C^(II)^ | 335.15  340.45 | 6.23 | Pd^0^ | 855.80  855.03 | 0.35 | Ni(OH)_2_ | 158.02  163.32 | 0.63 | Bi^0^ | **82** | **8** | **10** |
|  | 336.53  341.83 | 1.23 | PdO | 857.80  860.59 | 0.03 | Ni(OH)_2_ | 159.29  164.59 | 0.23 | Bi_2_O_3_ |  |  |  |
|  |  |  |  | 861.59  866.66 | 0.28 | Ni(OH)_2_ |  |  |  |  |  |  |
| Pd_85_Ni_10_Bi_5_/C^(III)^ | 335.15  340.45 | 4.61 | Pd^0^ | 855.79  855.02 | 0.57 | Ni(OH)_2_ | 157.99  163.29 | 0.47 | Bi^0^ | **72** | **17** | **10** |
|  | 336.53  341.83 | 0.27 | PdO | 857.79  860.58 | 0.04 | Ni(OH)_2_ | 159.33  164.63 | 0.17 | Bi_2_O_3_ |  |  |  |
|  |  |  |  | 861.58  866.66 | 0.46 | Ni(OH)_2_ |  |  |  |  |  |  |


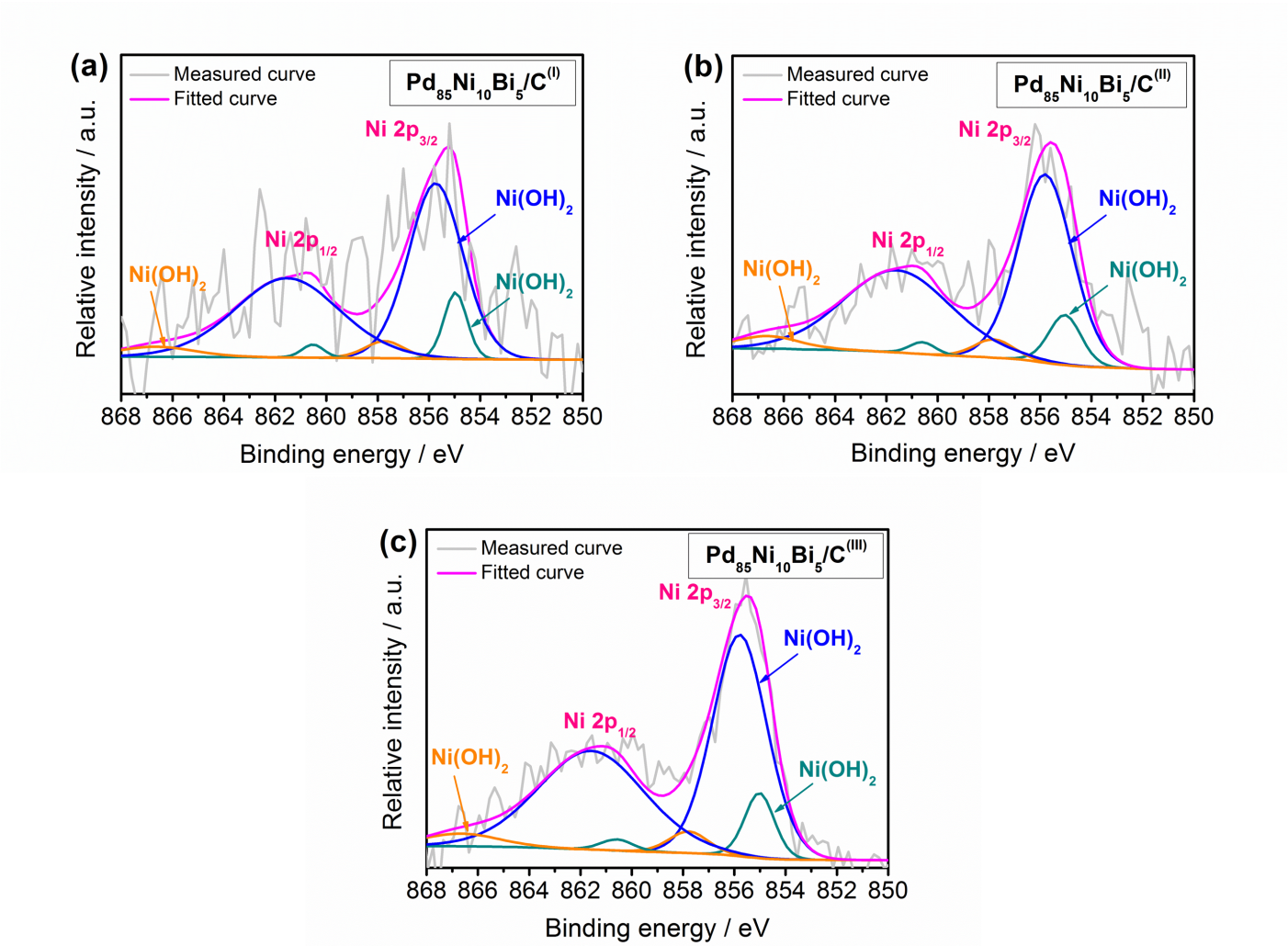


Fig. S8 Ni 2p core level XPS spectra of the (a) Pd_85_Ni_10_Bi_5_/C^(I)^, (b) Pd_85_Ni_10_Bi_5_/C^(II)^ and (c) Pd_85_Ni_10_Bi_5_/C^(III)^ catalyst


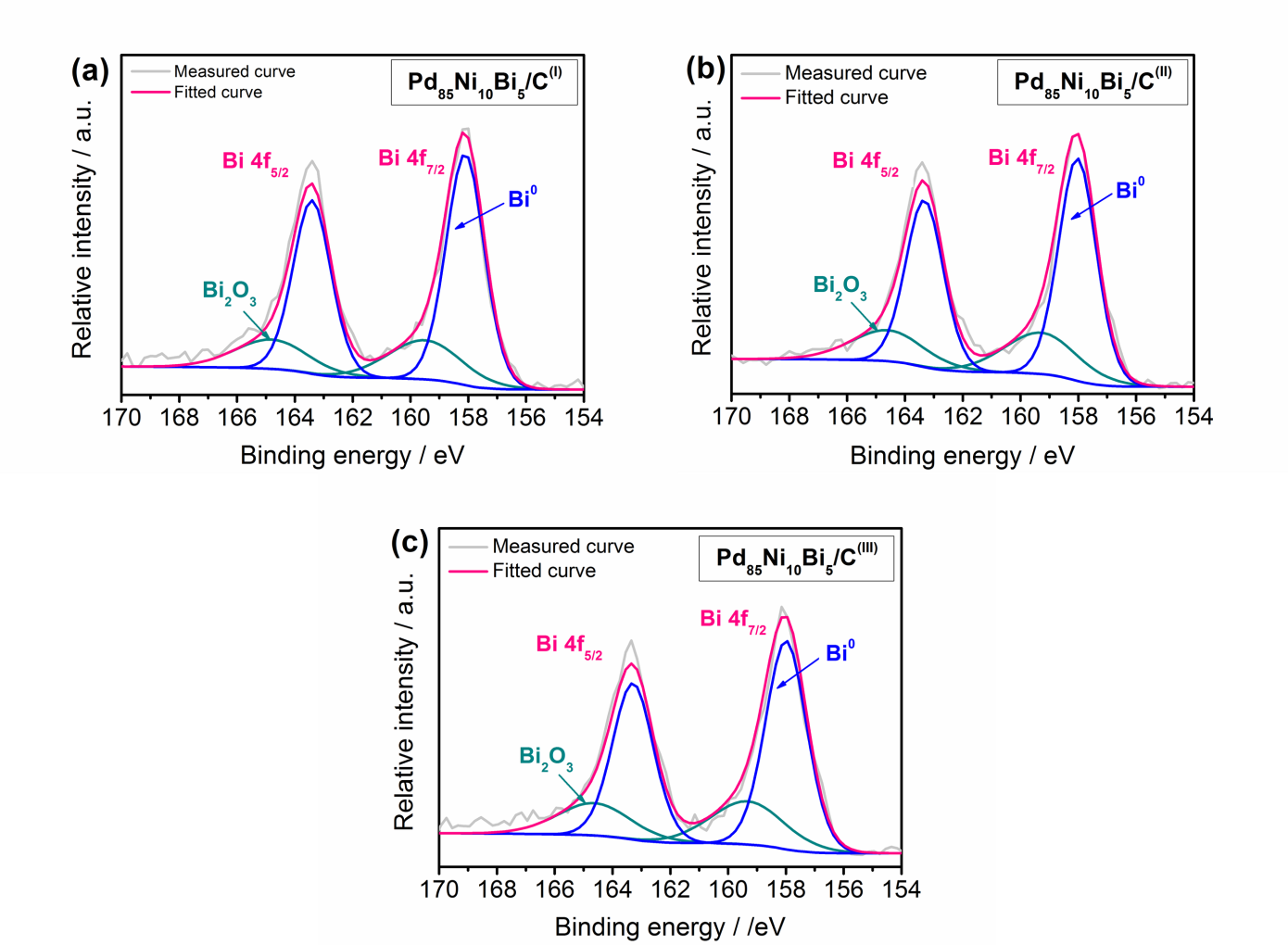


Fig. S9 Bi 4f core level XPS spectra of the (a) Pd_85_Ni_10_Bi_5_/C^(I)^, (b) Pd_85_Ni_10_Bi_5_/C^(II)^ and (c) Pd_85_Ni_10_Bi_5_/C^(III)^ catalyst







Fig. S10 CVs of (a) Pd_85_Ni_10_Bi_5_/C catalysts in de-aerated 1.0 M KOH at 30 °C and with a scan rate of 10 mV s^-1^ in the potential range of 0.05-1.5 V with (b) their resulting determined EASA

**Active layer thickness determination of the catalysts**

The active layer thickness (ALT) of the catalysts (commercial Pd/C and Pd_85_Ni_10_Bi_5_/C^(I)-(III)^) on the glassy carbon (GC) of the rotating disk electrode (RDE) was calculated according to the equations below assuming a uniformly distributed layer.

- **Steps for calculation of the ALT from the commercial Pd/C catalyst:**

$$m_{Pd}=\frac{m_{catalyst}}{V_{mixture}} \cdot V_{catalyst ink}\cdot0.4= \frac{0.0137 g}{5 mL} \cdot0.01 mL\cdot0.4= \boldsymbol{1.1 \cdot}\boldsymbol{10}^{\boldsymbol{-5}}\boldsymbol{g}$$

$$m_{Vulcan XC72}=\frac{m_{catalyst}}{V_{mixture}} \cdot V_{catalyst ink}\cdot0.6= \frac{0.0137 g}{5 mL} \cdot0.01 mL\cdot0.6= \boldsymbol{1.6 \cdot}\boldsymbol{10}^{\boldsymbol{-5}}\boldsymbol{g}$$

$$m_{Nafion 5 wt.\%}= \frac{\rho_{Nafion 5 wt.\%} \cdot V_{Nafion 5 wt.\%}\cdot\frac{5}{100}}{\frac{V_{mixture}}{V_{catalyst ink}}} = \frac{0.94 g\cdot{cm}^{-3} \cdot0.023 {cm}^{3} \cdot\frac{5}{100}}{\frac{5 mL}{0.01 mL}}= \boldsymbol{2.2 \cdot}\boldsymbol{10}^{\boldsymbol{-6}}\boldsymbol{g}$$

| $\boldsymbol{ALT}_{\boldsymbol{catalyst}}= \frac{{\frac{m_{Pd}}{\rho_{Pd}}}+ \frac{m_{Vulcan XC72}}{\rho_{Vulcan XC72}} + \frac{m_{Nafion 5 wt.\%}}{\rho_{Nafion}}}{A_{GC}}= \frac{{\frac{1.1 \cdot{10}^{-5} g}{12.02 g\cdot{cm}^{-3}}}+ \frac{1.6 \cdot{10}^{-5} g}{0.264 g\cdot{cm}^{-3}} + \frac{2.2 \cdot{10}^{-6} g}{1.98 g\cdot{cm}^{-3}}}{0.196 {cm}^{2}}= \boldsymbol{0.000328 cm=3.3 \mu m}$ | (9) |
| --- | --- |

- **Steps for calculation of the ALT from the Pd_85_Ni_10_Bi_5_/C^(I)-(III)^ catalysts:**

$$m_{Pd}=\frac{m_{catalyst}}{V_{mixture}} \cdot V_{catalyst ink}\cdot0.4= \frac{0.0162 g}{5 mL} \cdot0.01 mL\cdot0.4= \boldsymbol{1.3 \cdot}\boldsymbol{10}^{\boldsymbol{-5}}\boldsymbol{g}$$

$$m_{Vulcan XC72R}=\frac{m_{catalyst}}{V_{mixture}} \cdot V_{catalyst ink}\cdot0.6= \frac{0.0162 g}{5 mL} \cdot0.01 mL\cdot0.6= \boldsymbol{1.9 \cdot}\boldsymbol{10}^{\boldsymbol{-5}}\boldsymbol{g}$$

$$m_{Nafion 5 wt.\%}= \frac{\rho_{Nafion 5 wt.\%} \cdot V_{Nafion 5 wt.\%}\cdot\frac{5}{100}}{\frac{V_{mixture}}{V_{catalyst ink}}} = \frac{0.94 g\cdot{cm}^{-3} \cdot0.023 {cm}^{3} \cdot\frac{5}{100}}{\frac{5 mL}{0.01 mL}}= \boldsymbol{2.2 \cdot}\boldsymbol{10}^{\boldsymbol{-6}}\boldsymbol{g}$$

| $\boldsymbol{A}\boldsymbol{LT}_{\boldsymbol{catalyst}}= \frac{{\frac{m_{Pd}}{\rho_{Pd}}}+ \frac{m_{Vulcan XC72R}}{\rho_{Vulcan XC72R}} + \frac{m_{Nafion 5 wt.\%}}{\rho_{Nafion}}}{A_{GC}}= \frac{{\frac{1.3 \cdot{10}^{-5} g}{12.02 g\cdot{cm}^{-3}}}+ \frac{1.9 \cdot{10}^{-5} g}{0.096 g\cdot{cm}^{-3}} + \frac{2.2 \cdot{10}^{-6} g}{1.98 g\cdot{cm}^{-3}}}{0.196 {cm}^{2}}= \boldsymbol{0.001044 c}\boldsymbol{m=10.4 \mu m}$ | | (10) | |
| --- | --- | --- | --- |
| ${ALT}_{catalyst}$ | Active layer thickness (= Pd + Nafion ionomer (5 wt.%, Quintech) as binder + Vulcan) of the catalyst used | |  |
| $\frac{m_{catalyst}}{V_{mixture}}$ | Catalyst ink:  A certain amount of the catalyst (m_catalyst_ = 13.7 mg for comm. Pd/C; m_catalyst_ = 16.2 mg for Pd_85_Ni_10_Bi_5_/C^(I-III)^) in a mixture of  3477 µL 2-propanol, 1500 µL ultra-pure water and 23 µL Nafion ionomer solution (V_mixture_ = 5 mL) | |  |
| $V_{catalyst ink}$ | 10 µL of the catalyst ink was applied onto the GC disk to achieve the loading of 56 µg_Pd_ cm^-2^ for the catalyst used | |  |
| $V_{Nafion 5 wt.\%}$ | 23 µL (= 0.023 cm^3^) of Nafion ionomer solution | |  |
| $m_{Pd}=\frac{m_{catalyst}}{V_{mixture}} \cdot V_{catalyst ink}\cdot0.4$ | Mass of Pd (40 wt.% of the catalyst) | |  |
| $m_{Vulcan}=\frac{m_{catalyst}}{V_{mixture}} \cdot V_{catalyst ink}\cdot0.6$ | Mass of Vulcan XC72 (60 wt.% of Pd/C)  Mass of Vulcan XC72R (60 wt.% of Pd_85_Ni_10_Bi_5_/C^(I-III)^) | |  |
| $m_{Nafion 5 wt.\%}= \frac{\rho_{Nafion 5 wt.\%} \cdot V_{Nafion 5 wt.\%}\cdot\frac{5}{100}}{\frac{V_{mixture}}{V_{catalyst ink}}}$ | Mass of Nafion ionomer | |  |
| $V_{Pd}=\frac{m_{Pd}}{\rho_{Pd}}$ | Volume of Pd | |  |
| $V_{Vulcan}=\frac{m_{Vulcan}}{\rho_{Vulcan}}$ | Volume of Vulcan XC72 (Pd/C)  Volume of Vulcan XC72R (Pd_85_Ni_10_Bi_5_/C^(I-III)^) | |  |
| $V_{Nafion 5 wt.\%}=\frac{m_{Nafion 5 wt.\%}}{\rho_{Nafion}}$ | Volume of Nafion ionomer | |  |
| $\rho_{Nafion 5wt.\%}$ | Density of Nafion ionomer (0.94 g cm^-3^) | |  |
| $\rho_{Pd}$ | Density of Pd (Lit.: 12.02 g cm^-3^)  <https://www.reade.com/products/palladium-pd-powder-shot-pellets-ingot> | |  |
| $\rho_{Vulcan XC72}$ | Density of Vulcan XC72 (Lit.: 0.264 g cm^-3^)  <https://fuelcellstore.com/fuel-cell-components/vulcan-xc72> | |  |
| $\rho_{Vulcan XC72R}$ | Density of Vulcan XC72R (Lit.: 0.096 g cm^-3^)  <https://fuelcellstore.com/fuel-cell-components/vulcan-xc-72r> | |  |
| $\rho_{Nafion}$ | Density of Nafion (Lit.: 1.98 g cm^-3^)  <http://www.nafionstore.com/Shared/Bulletins/N115-N117-N1110.pdf> | |  |
| $A_{GC-RDE}$ | Area of the GC-RDE (Ø = 5 mm; 0.196 cm^2^) | |  |

The thickness of the catalyst layer has an influence on mass transport and diffusion. It has previously been shown in a detailed study how the thickness of the catalyst affects electrochemical measurements [43]. The variation of the electrode thickness was outside the scope of this manuscript.

**Reference**

43. K.J.J. Mayrhofer, D. Strmcnik, B.B. Blizanac, V. Stamenkovic, M. Arenz, N.M. Markovic, Measurement of oxygen reduction activities via the rotating disc electrode method: From Pt model surfaces to carbon-supported high surface area catalysts. Electrochim. Acta **53**, 3181–3188 (2008)
